# Comparative genomic analysis of ESBL-producing *Escherichia coli* from faecal carriage and febrile urinary tract infection in children: a prospective multicentre study

**DOI:** 10.1093/jacamr/dlac056

**Published:** 2022-05-21

**Authors:** Philippe Bidet, André Birgy, Naim Ouldali, Stéphane Béchet, Corinne Levy, Fouad Madhi, Elsa Sobral, Robert Cohen, Stéphane Bonacorsi

**Affiliations:** Université Paris Cité, IAME, INSERM, F-75018 Paris, France; Service de Microbiologie, Centre National de Référence associé pour Escherichia coli, Hôpital Robert-Debré, AP-HP, Paris, France; Université Paris Cité, IAME, INSERM, F-75018 Paris, France; Service de Microbiologie, Centre National de Référence associé pour Escherichia coli, Hôpital Robert-Debré, AP-HP, Paris, France; Association Clinique Thérapeutique Infantile du Val de Marne (ACTIV), Créteil, France; Service de Pédiatrie Générale, Hôpital Robert-Debré, AP-HP, Paris, France; Association Clinique Thérapeutique Infantile du Val de Marne (ACTIV), Créteil, France; Association Clinique Thérapeutique Infantile du Val de Marne (ACTIV), Créteil, France; Université Paris Est, IMRB-GRC GEMINI, Créteil, France; GPIP (Groupe de Pathologie Infectieuse Pédiatrique) de la SFP (Société Française de Pédiatrie), Paris, France; Association Clinique Thérapeutique Infantile du Val de Marne (ACTIV), Créteil, France; Université Paris Est, IMRB-GRC GEMINI, Créteil, France; GPIP (Groupe de Pathologie Infectieuse Pédiatrique) de la SFP (Société Française de Pédiatrie), Paris, France; Service de Pédiatrie Générale, Centre Hospitalier Intercommunal de Créteil, Créteil, France; Association Clinique Thérapeutique Infantile du Val de Marne (ACTIV), Créteil, France; Association Clinique Thérapeutique Infantile du Val de Marne (ACTIV), Créteil, France; Université Paris Est, IMRB-GRC GEMINI, Créteil, France; GPIP (Groupe de Pathologie Infectieuse Pédiatrique) de la SFP (Société Française de Pédiatrie), Paris, France; Université Paris Cité, IAME, INSERM, F-75018 Paris, France; Service de Microbiologie, Centre National de Référence associé pour Escherichia coli, Hôpital Robert-Debré, AP-HP, Paris, France

## Abstract

**Background:**

The reliability of ESBL-producing *Escherichia coli* (ESBL-Ec) faecal carriage monitoring to guide probabilistic treatment of febrile urinary tract infection (FUTI) in children remains unclear.

**Objectives:**

To compare the genomic characteristics of ESBL-Ec isolates from faecal carriage and FUTI to assess their correlation and identify a FUTI-associated virulence profile.

**Methods:**

We conducted a prospective multicentre hospital and ambulatory-based study. We analysed the genotypes and virulence factors of both faecal and FUTI ESBL-Ec by whole genome sequencing. Correlations were assessed by non-parametric Spearman coefficient and virulence factors were assessed by chi-squared tests with Bonferroni correction.

**Results:**

We included 218 ESBL-Ec causing FUTI and 154 ESBL-Ec faecal carriage isolates. The most frequent ST was ST131 (44%) in both collections. We found high correlation between carriage and ESBL-Ec FUTI regarding genes/alleles (rho = 0.88, *P *< 0.0001) and combinations of virulence genes, MLST and serotypes (rho = 0.90, *P *< 0.0001, rho = 0.99, *P *= 0.0003, rho = 0.97, *P *= 0.005 respectively). Beside this strong correlation, we found five genes that were significantly associated with FUTI (*papC*, *papGII*, *hlyC*, *hek* and *traJ*). The strongest association with FUTI was found with adhesin gene allele *papGII* (54% in FUTI versus 16% in carriage) and for *papGII* and gene *traJ* alone or in combination (63% versus 24%).

**Conclusions:**

The genomic profile of ESBL-Ec causing FUTI in children strongly correlates with faecal carriage isolates except for a few genes. The presence of *papGII* and/or *traJ* in a previously identified carriage strain could be used as a marker of uropathogenicity and may guide the empirical antimicrobial choice in subsequent FUTI.

## Introduction

Both antimicrobial resistance and extraintestinal virulence factors have increased in *Escherichia coli* faecal carriage isolates over the last decades.^1^ However, the link between virulence and resistance in *E. coli* has raised many debates and controversy. While several studies have reported that resistance to quinolones was associated with less-virulent strains,^2^ or that highly virulent clonal groups harboured fewer resistance determinants,^3^ the recent ESBL pandemic is linked to a sequence type ST131 belonging to the virulent phylogenetic group B2.^4^ In this study we have focused on the ESBL-producing *E. coli* (ESBL-Ec) strains isolated in children both in community-acquired febrile urinary tract infections (FUTI) and during asymptomatic faecal carriage to compare their virulomes.

In children, as in adults, *E. coli* is by far the most frequent aetiological agent of FUTI both in hospitalized patients and in the community.^5^ Pathophysiology of FUTI begins with urethral colonization by uropathogenic *E. coli* (UPEC) strains issuing from the gut microbiota.^6^ Strains causing pyelonephritis harbour various virulence factors facilitating ascending colonization of the urinary tract.^6^

Pyelonephritis is particularly harmful in young children, and can be associated with bacteraemia and renal scarring, which may eventually be complicated by chronic high blood pressure or renal failure.^7,8^ Delayed antimicrobial therapy in FUTI is associated with adverse outcome.^8^

As about 50% of strains produce class A β-lactamases, empirical antimicrobial therapy of *E. coli* pyelonephritis mainly consists of either third-generation cephalosporins and/or aminoglycosides.^9^ Resistance to those antibiotics is increasing worldwide both in adults and children, mainly due to the emergence of ESBL-producing strains.^10–12^ The increasing percentage of ESBL-producing strains thus questions empirical antimicrobial choice.

However, implementing carbapenems as first line antibiotics for treatment of this common infection would lead to an increase in carbapenem-resistant strains in children, with adverse consequences for future patients.^13^ Rapid tests (either phenotypic or genetic) able to screen for ESBL directly in urine can be used to guide antimicrobial choice but have some drawbacks (such as unavailability at the time of diagnosis, or contamination by a few ESBL-producing bacteria not involved in the infectious process).^14,15^

Faecal carriage monitoring of resistant strains has been proposed to adapt empirical antimicrobial therapy before drug susceptibility testing (DST) results on infecting bacteria are available, especially for patients particularly at risk of invasive infection such as patients with known urologic disorder, premature infants or immunocompromised children.^16^ This strategy relies on the hypothesis that ESBL-Ec causing infections correlate with ESBL-Ec carriage isolates, a postulate which remains to be further confirmed.

Unfortunately, these patients, who are often hospitalized for long periods of time, are also more frequently carriers of multidrug-resistant bacteria and thus more frequently treated with broad-spectrum β-lactams.^17^ A method allowing the detection among resistant faecal carriage strains of those more likely to cause FUTI would be helpful to focus effective antibiotics against these strains on patients carrying them.

In this study, we first assessed the genomic correlation between ESBL-Ec faecal carriage and ESBL-Ec causing community-acquired FUTI. We then aimed to identify a FUTI-associated virulence profile by detecting genomic characteristics of *E. coli* isolates significantly associated with FUTI.

## Material and methods

### Study design, patients and bacteria

We conducted a prospective ambulatory and hospital-based multicentre study. We collected paediatric community-acquired FUTI cases caused by ESBL-Ec from March 2014 to March 2017 in a French tertiary hospital as previously described.^11,18^ From October 2010 to July 2017, eight French ambulatory paediatricians located in three regions (Ile de France, Lorraine and Provence-Alpes-Côte d'Azur) took part in a prospective study analysing faecal carriage of ESBL-Ec among children aged 6 to 24 months in the community as previously described.^10,19^ All isolates from faecal carriage and FUTI were whole genome sequenced. Some of the isolates found in FUTI have been previously described globally to objectify the diversity and trends in ESBL-producing Enterobacterales found in FUTI in children. However, their virulence content has not been studied exhaustively and they had not been compared with faecal isolates.^11^

### Whole-genome sequencing and genotyping

The Nextera XT kit (Illumina, USA) was used to prepare libraries. Sequencing was performed on a HiSeq instrument for 2 × 100 cycles (Illumina Technology). The SPAdes assembler was used to construct assemblies. The quality of the sequencing data was estimated using standard metrics including N50 and coverage. Identification of acquired ESBL genes was performed using ResFinder 4.1 and MLST for *E. coli* (Warwick scheme).^20,21^ We also determined serotype, *fimH* allele and phylogenetic groups (SerotypeFinder 1.1, fimTyper and Enterobase).^22–24^ Within *E. coli* of ST131, we distinguished different clades as previously defined.^25–28^ Clade A is associated with *fimH41*, clade B with *fimH22* and clade C with *fimH30*. Within clade C, two subclades have been identified. The C1 subclade, also called ST131 H30R, comprises isolates with mutations in the chromosomal *gyrA* and *parC* genes, which confer resistance to fluoroquinolones. Subclade C1 was then separated between isolates harbouring *bla*_CTX-M-27_ (C1-M27) or not (C1nM27).^29^ The C2 subclade, also called ST131 H30-Rx, groups isolates with the same *gyrA* and *parC* mutations, and the *bla*_CTX-M-15_ gene.

Raw reads have been deposited in GenBank under BioProject PRJNA551371 for FUTI isolates,^11^ and under BioProjects PRJNA522367^19^ for faecal carriage isolates (under completion).

### Virulence factors

Virulence factor genes were identified using the NCBI BLAST tool (BLAST version 2.2.31) to search for 180 genes and alleles (Table S1, available as Supplementary data at *JAC-AMR* Online). A threshold of 90% for both coverage and identity was used. The presence of a putative pS88-like ColV plasmid was defined as co-occurrence of *iroD*, *iucC*, *sitA*, *hlyF* and *mig14*.^30^ Among putative pathogenicity islands (PAIs), PAI-II-J96/PAI-I-C5 was defined by the co-occurrence of *hlyC*, *papC*, *cnf1* and *hek*,^31^ PAI-I-CFT073 (pheV inserted) by *hlyC*, *papC*, *iucC*, *iha* and *sat*,^32^ PAI-III-536/PAI-CFT073-serX by *sfaS* and *iroD.*^33^

### Statistics

First, we analysed the correlation between genomic characteristics from faecal carriage isolates and ESBL-Ec strains causing FUTI using the non-parametric Spearman correlation coefficient. Correlation was assessed for genes/alleles, and then combination of genes, MLST and serotypes.

Second, we compared the proportion of each gene or allele among FUTI isolates versus carriage isolates using chi-squared tests. Bonferroni correction was used to avoid random false-positive associations among the 180 independent chi-squared tests performed for virulence genes. *P* values were in this case considered significant if <0.00028.

### Ethics

The data collection was approved by the French National Data Protection Commission (CNIL, no. 913582), the Committee on the Processing of Research Information (CCTIRS, no. 13.341) and the Ethics Committee of the Créteil Intercommunal Hospital. All legal guardians of included children provided oral informed consent. The study was registered at ClinicalTrials.gov (registration no. NCT02832258).

## Results

### Population characteristics

The median age was 13 months (IQR 10–17) for children from the carriage cohort and 12 months (IQR 4–30) for children from the FUTI cohort. A uropathy was identified in 42/218 (19%) children with FUTI.

The sex ratio was 0.80 (44.5% of male children) in the FUTI population and 0.92 (48.0% of male children) in the carriage population.

### Sequencing results

We included and whole genome sequenced 218 FUTI ESBL-Ec and 154 faecal carriage isolates.

Sequencing data are presented in Table S2. Mean coverage was 104× and mean N50 was 173* *714 bp.

### Sequence types, serotypes and fimH types

The most frequent molecular features of the strains are presented in Table 1, detailed molecular typing is presented in Table S1. The most frequent phylogroup was B2 both in FUTI (64%) and carriage isolates (60%) whereas the most frequent STs were ST131 (44%), ST38 (9%), ST69 (6%), ST73 (3%) and ST95 (3%) in both collections with similar rates. The most frequent serotypes were O25:H4 (38%), O16:H5 (8%), both of them associated with ST-complex (STc) 131, O6:H1 (2%), O75:H5 (3%) and O86:H18 (5%). Among the different clades and subclades of ST131, subclade C2H30Rx was associated with FUTI (27% versus 16%, *P *< 0.05) while clade C1 and subclade C1-M27 were associated with carriage isolates (21 and 18% versus 7 and 5%, respectively, *P *< 0.05). The most frequent *fimH* types encountered were *fimH30* (40%), *fimH27* (14%) and *fimH41* (9%). The percentage of *fimH27* strains was higher among FUTI strains than among carriage strains (18% versus 8%, *P *= 0.007). This particular *fimH27* was associated with various STs, the most frequent being ST69 (34%).

**Table 1.** Most frequent virulence-associated genes/alleles, genotypes and serotypes among febrile urinary tract infection (FUTI) and faecal carriage ESBL-producing *E. coli* isolates in children^a^

	Number and percentage of isolate groups							*P* value	
Characteristic	All isolates (*n *= 372)		FUTI isolates (*n *= 218)		Carriage isolates (*n *= 154)		Delta % (FUTI−carriage)	*χ* ^2^	*χ* ^2^ with Bonferroni correction
Single gene(s)/allele(s)									
*gad*	351	94%	205	94%	146	95%	−1%	NS	NS
*fyuA*	333	90%	202	93%	131	85%	8%	<0.05	NS
*chuA*	331	89%	200	92%	131	85%	7%	<0.05	NS
*sitA*	321	86%	196	90%	125	81%	9%	<0.05	NS
*kpsF*	312	84%	190	87%	122	79%	8%	<0.05	NS
*irp2*	287	77%	175	80%	112	73%	8%	NS	NS
*iss*	294	79%	175	80%	119	77%	3%	NS	NS
*iucC*	289	78%	174	80%	115	75%	5%	NS	NS
*iutA*	291	78%	174	80%	117	76%	4%	NS	NS
*shiF*	290	78%	174	80%	116	75%	4%	NS	NS
*ihA-like*	233	63%	144	66%	89	58%	8%	NS	NS
*sat*	225	60%	137	63%	88	57%	6%	NS	NS
*papC*	157	42%	126	58%	31	20%	38%	<0.05	<0.00028
*papGII*	141	38%	117	54%	24	16%	38%	<0.05	<0.00028
*imm*	194	52%	116	53%	78	51%	3%	NS	NS
*senB*	175	47%	105	48%	70	45%	3%	NS	NS
*hek*	134	36%	97	44%	37	24%	20%	<0.05	<0.00028
*hlyC*	93	25%	74	34%	19	12%	22%	<0.05	<0.00028
*traJ*	80	22%	62	28%	18	12%	17%	<0.05	<0.00028
*K2-type kfiA*	75	20%	57	26%	18	12%	14%	<0.05	NS
*eilA*	88	24%	56	26%	32	21%	5%	NS	NS
*cnf1*	70	19%	52	24%	18	12%	12%	<0.05	NS
*nfaE*	74	20%	50	23%	24	16%	7%	NS	NS
*aafC*	72	19%	48	22%	24	16%	6%	NS	NS
*iroD*	74	20%	45	21%	29	19%	2%	NS	NS
*astA*	66	18%	44	20%	22	14%	6%	NS	NS
*K5*-type *KfiA*	87	23%	43	20%	44	29%	−9%	<0.05	NS
Combinations									
*papGII* and/or *hlyC* and/or *traJ*	187	50%	143	66%	44	29%	37%	<0.05	<0.00028
*papC* and/or *traJ*	185	50%	142	65%	43	28%	37%	<0.05	<0.00028
*papC* and/or *hek*	194	52%	141	65%	53	34%	30%	<0.05	<0.00028
*papGII* and/or *hek*	192	52%	139	64%	53	34%	29%	<0.05	<0.00028
*papGII* and/or *hlyC* and/or *hek*	192	52%	139	64%	53	34%	29%	<0.05	<0.00028
*papGII* and/or *traJ*	174	47%	137	63%	37	24%	39%	<0.05	<0.00028
*papC* and/or *K2*	175	47%	133	61%	42	27%	34%	<0.05	<0.00028
*papGII* and/or *hlyC* and/or *K2*	172	46%	130	60%	42	27%	32%	<0.05	<0.00028
*papC* and/or *hlyC*	160	43%	128	59%	32	21%	38%	<0.05	<0.00028
*papC* and/or *cnf1*	160	43%	128	59%	32	21%	38%	<0.05	<0.00028
*papGII* and/or *K2*	160	43%	125	57%	35	23%	35%	<0.05	<0.00028
*papGII* and/or *hlyC*	154	41%	123	56%	31	20%	36%	<0.05	<0.00028
*papGII* and/or *cnf1*	154	41%	123	56%	31	20%	36%	<0.05	<0.00028
*papGII* and/or *hlyC* and/or *cnf1*	154	41%	123	56%	31	20%	36%	<0.05	<0.00028
Putative pathogenicity island/plasmid pS88									
PAI I CFT073 (*hlyC, papC, iucC, iha-like, sat*)	110	30%	89	41%	21	14%	27%	<0.05	<0.00028
PAI IIJ96/PAI IC5 (*hlyC, papC, cnf1, hek*)	54	15%	42	19%	12	8%	11%	<0.05	NS
PAI III536 (*sfaS, iroD*)	9	2%	5	2%	4	3%	0%	NS	NS
pS88 (*iroD, iucC, sitA, hlyF, mig14, traJ*)	27	7%	16	7%	11	7%	0%	NS	NS
FimH types									
*fimH30*	147	40%	84	39%	63	41%	−2%	NS	
*fimH27*	53	14%	40	18%	13	8%	10%	<0.05	
*fimH41*	34	9%	17	8%	17	11%	−3%	NS	
MLST (Warwick scheme)									
ST131	164	44%	92	42%	72	47%	−5%	NS	
ST38	32	9%	23	11%	9	6%	5%	NS	
ST69	21	6%	15	7%	6	4%	3%	NS	
ST73	13	3%	9	4%	4	3%	2%	NS	
ST95	12	3%	9	4%	3	2%	2%	NS	
phylogroup B2	232	62%	140	64%	92	60%	4%	NS	
Serotype									
O25:H4	141	38%	80	37%	61	40%	−3%	NS	
O16:H5	31	8%	17	8%	14	9%	−1%	NS	
O86:H18	20	5%	11	5%	9	6%	−1%	NS	
O75:H5	10	3%	7	3%	3	2%	1%	NS	
O6:H1	9	2%	6	3%	3	2%	1%	NS	
ESBL genes									
*bla*_CTX-M-15_	161	43%	114	52%	47	31%	21%	<0.05	
*bla*_CTX-M-14_	72	19%	39	18%	33	21%	−3%	NS	
*bla*_CTX-M-27_	71	19%	31	14%	40	26%	−12%	<0.05	
*bla*_CTX-M-1_	44	12%	17	8%	27	18%	−10%	<0.05	
Subclades of ST131									
Clade A	25	7%	15	7%	10	6%	1%	NS	
C1	48	13%	15	7%	33	21%	−14%	<0.05	
C1-M27	37	10%	10	5%	27	18%	−13%	<0.05	
C1-nM27	11	3%	5	2%	6	4%	−2%	NS	
C2/H30Rx	83	22%	58	27%	25	16%	11%	<0.05	

a For each gene/allele or combination of genes, the number and percentage of isolates harbouring this attribute is indicated. The Delta % is calculated as the percentage in FUTI isolates minus the percentage in faecal carriage isolates. Chi squared (*χ*^2^) *P* value is indicated without and with Bonferroni correction. NS, non-significant.

### Genomic correlation between E. coli faecal carriage and ESBL-Ec causing FUTI

**Figure 1.**
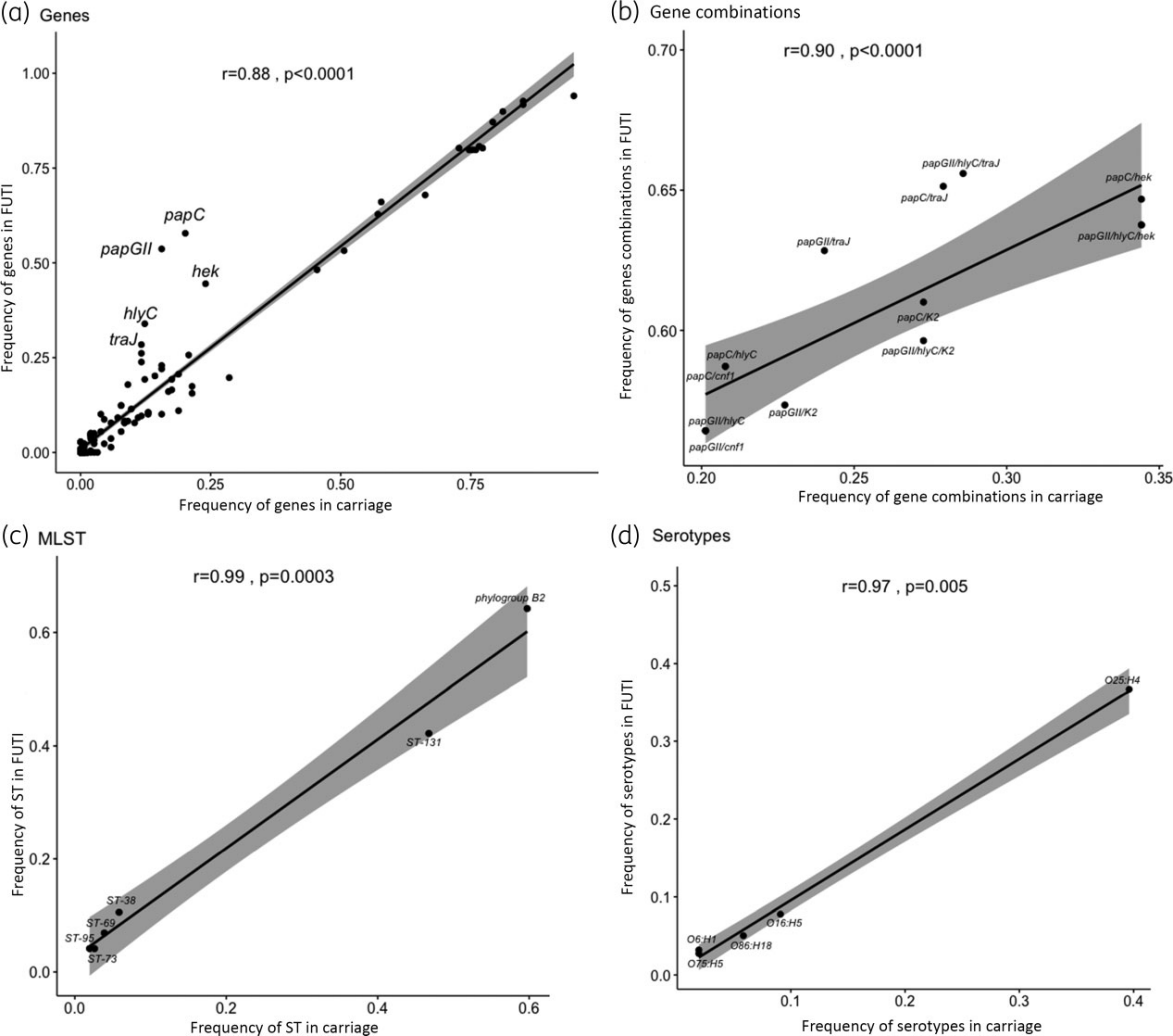
Correlation between ESBL-producing *E. coli* from faecal carriage and febrile urinary tract infection (FUTI) in children (*N *= 372). (a) Depending on genes. (b) Depending on gene combinations. (c) Depending on MLST. (d) Depending on serotypes.

We found a very high correlation between carriage and ESBL-Ec FUTI regarding the 180 genes/alleles associated with *E. coli* virulence (Table S1) (rho = 0.88, *P *< 0.0001, Figure 1a).

Then, we confirmed this highly significant correlation between carriage and ESBL-Ec FUTI when analysing combinations of virulence genes (rho = 0.90, *P *< 0.0001), MLST (rho = 0.99, *P *= 0.0003), and serotypes (rho = 0.97, *P *= 0.005 respectively, Figure 1b–d).

### Putative virulence genes

Among the 180 genes associated with *E. coli* virulence, 27 were found in more than 20% of FUTI isolates (Table 1). Most of them were genes involved in iron capture (yersiniabactin: *fyuA* and *irp2*; salmochelin: *iroD*; aerobactin: *iucC* and *iutA*, *chuA*, *sitA*); capsule biosynthesis (*kpsf*, *KfiA*); adhesion (type P-pili: *papC* and *papGII*, *ihA*, *eilA*, *nfaE*, *aafC*); invasion (*hek*) or cell toxicity (*hlyC*, *cnf1*, *sat*, *senB*).

After Bonferroni correction, only 5 of the 180 putative or known virulence genes and alleles searched (*papC*, *papGII*, *hlyC, hek* and *traJ*) remained significantly associated with FUTI (Table 1 and Table S1). All *papGII*-positive isolates were also *papC* positive, both genes belonging to the *pap* operon. The strongest association with FUTI was found for PapG adhesin gene allele *papGII* (54% versus 16%, *P *= 8.8079E−14). Among FUTI cases *papGII* was present in 48% of those with a known urologic disorder versus 55% of those without any (*P *= 0.3597, non-significant). Adhesin *papGII* was found in 75% of *fimH27* FUTI isolates versus 23% of *fimH27* carriage isolates.

We tested the possibility to combine *papGII* with the other genes (except *papC*) also associated with urinary virulence (Table 1 and Figure 1b) in order to increase the percentage of FUTI isolates categorized with a risk genotype, a low-risk isolate being an isolate without any of the markers.

The strongest association of gene combinations with FUTI was found with the combination of *papGII* and/or *traJ*: isolates harbouring at least one of these two genes represented 63% of FUTI cases versus 24% of healthy carriage cases. This dual detection used as a screening test would thus have a sensitivity of 63%, a specificity of 76%, a positive predictive value of 79% and a negative predictive value of 59% (Figure S1).

### Combinations of genes suggesting the presence of putative PAIs and virulence plasmid

The presence of a putative pS88-like ColV plasmid (defined as co-occurrence of *iroD, iucC, sitA, hlyF* and *mig14*) was found in about 10% of strains without significant difference between FUTI and carriage. Among putative PAIs, PAI-I-CFT073 (defined as the co-occurrence of *hlyC, papC, iucC, iha-like* and *sat*) was significantly more frequent among FUTI isolates than among carriage isolates (41% versus 14%, *P *< 0.01). PAI-II-J96/PAI-I-C5 (defined as the co-occurrence of *hlyC, papC, cnf1* and *hek*) was found in 19% of FUTI isolates versus 8% of carriage isolates, however this difference did not remain significant after Bonferroni correction.

### ESBL genes

The most frequent ESBL genes encountered among the whole collection of isolates (FUTI and carriage) were *bla*_CTX-M-15_ (*n *= 161, 43%), *bla*_CTX-M-14_ (*n *= 72, 19%), *bla*_CTX-M-27_ (*n *= 71, 19%) and *bla*_CTX-M-1_ (*n *= 44, 12%).

*bla*_CTX-M-15_ was more frequent among FUTI isolates (52% versus 31%, *P *< 0.01), while *bla*_CTX-M-27_ and *bla*_CTX-M-1_ were less frequently encountered than among carriage isolates (8% versus 18 and 14% versus 26% respectively, *P *< 0.01).

Whatever the origin, isolates carrying *bla*_CTX-M-15_ were more frequently equipped with PapGII adhesin (55%) than those carrying either *bla*_CTX-M-1_ (11%) or *bla*_CTX-M-27_ (18%), *P *< 0.01.

The gene *traJ* was present in 36.4% of *bla*_CTX-M-1_, 19.4% of *bla*_CTX-M-14_, 26.3% of *bla*_CTX-M-15_ and 1.4% of *bla*_CTX-M-27_ isolates.

### Comparison of O25:H4 and O16:H5 subclones among STc-131 isolates

Among STc131 isolates of the whole collection, those belonging to O25:H4 serotype (*n *= 141) more frequently harboured PapGII adhesin and *bla*_CTX-M-15_ β-lactamase gene than O16:H5 serotype (*n *= 31) (50% versus 26%, *P *< 0.05 and 63% versus 26%, *P *< 0.05, respectively) (Table 2)

**Table 2.** Comparison of O16:H5 and O25:H4 among STc-131 isolates

	Number and percentage of isolates					
All STc-131 isolates	O16:H5 (*n *= 31)		O25:H4 (*n *= 141)		Delta %	*χ* ^2^ *P* value
*papGII*	8	26%	70	50%	−24%	<0.05
*bla*_CTX-M-14_	6	19%	11	8%	12%	NS
*bla*_CTX-M-15_	8	26%	89	63%	−37%	<0.05
*bla*_CTX-M-27_	13	42%	40	28%	14%	NS

### Comparison of St131 clades and subclades

*bla*_CTX-M-15_ was present in 32% of clade A isolates, none of the C1 isolates, and 100% of C2 isolates. The gene *papGII* was present in 28% of clade A isolates, 16.7% of C1 isolates, and 68.7% of C2 isolates. The gene *traJ* was absent in clade A and C1 isolates but present in 36.1% of C2 isolates.

## Discussion

In this study we have compared the genomes of ESBL-Ec isolates from children with either healthy gut carriage or FUTI. We first found a strong correlation between the genomics of ESBL-Ec faecal carriage and ESBL-Ec causing FUTI. This similar genomic profile between carriage and infection has been recently suggested by Verschuuren *et al.*,^34^ who found that the distributions of the 10 most prevalent genes from ESBL-Ec faecal carriage and extra-intestinal infection often overlapped. Taken together, these findings suggest that faecal carriage monitoring may be a valuable tool to monitor ESBL-Ec strains involved in FUTI.

Beside this high correlation, we identified some particular bacterial traits associated with invasive infection. A few genes were significantly associated with ESBL-Ec FUTI. After Bonferroni’s adjustment, *papGII*, *papC*, *hlyC*, *hek* and *traJ* were positively associated with a risk of FUTI among ESBL-Ec isolates. All those five genes or gene alleles have been previously associated with *E. coli* extra-intestinal virulence, implicated at different steps of the pathogenesis process.

Gene *papC* and the *papGII* allele of gene *papG* belong to the same operon encoding type P pilus. Thus, both genes are physically linked and all *papGII-*positive strains are also *papC* positive. PapC is a conserved outer-membrane protein acting as a molecular usher in type P pilus assembly while PapG mediates adhesion to urothelial globoside’s (α-gal-1-4 β-gal) disaccharide.^35^ Several alleles of adhesin PapG exist with different tropisms. PapGII is the allele specifically involved in the pathogenesis of pyelonephritis. *E. coli* strains lacking this allele rarely cause pyelonephritis except in case of pre-existing urinary tract abnormalities such as urinary tract obstruction, anatomical abnormalities or vesicoureteral reflux.^6^ Catheter-associated bacteriuria isolates are also less likely to harbour this virulence factor.^36^ In our population, a urologic disorder was documented in 23% of FUTI cases and *papGII* was slightly less frequent in those children (48%) than in those without known abnormality (55%), but this difference was not significant.^18^ Thus, the low percentage of *papGII*-positive isolates in our collection of ESBL-Ec isolates causing FUTI, contrasting with the higher percentages (70%–80%) usually observed in other studies on pyelonephritis in children,^6^ could not be explained by a higher rate of urinary tract impairment. However, the clinical data that were collected concerned only already known disorders at the time of infection and not those that could have been discovered following this episode of FUTI, and the actual percentage of urologic disorders may in fact have been higher.

The presence of *papGII* was significantly more frequent among ESBL-Ec isolates carrying the *bla*_CTX-M-15_ gene than among those carrying *bla*_CTX-M-1_ or *bla*_CTX-M-27_, thus explaining the association of *bla*_CTX-M-15_ with FUTI. Among STc-131 isolates, this link between *papGII* and *bla*_CTX-M-15_ was related to the emergent O25:H4 serotype frequently harbouring both attributes^11^ and more precisely subclade C2/H30Rx associated with FUTI isolates (Table 1).

Gene *hlyC* is part of the *hlyCABD* operon involved in α-haemolysin synthesis. Its exact role in FUTI is still unclear and probably multifactorial, including lysis of white blood cells (such as natural-killer cells) and epithelial cells.^37^ The *hly* operon is frequently collocated with *pap* operon within pathogenicity islands specific for UPEC strains.^36^ Thus, the combinations including *hly* and *pap* operons such as those suggesting the presence of a putative PAI I_CFT073_ or PAI II_J96_/PAI I_C5_ (Table 1) were also significantly associated with FUTI isolates. This fact may explain why combining the detection of the *hlyC* gene with either *papC* or *papGII* did not significantly increase the number of strains carrying at least one of those genes.

Hek outer membrane protein is an auto-aggregating adhesin and invasin initially described in neonatal meningitis *E. coli* (NMEC) isolates of capsular serogroup K1.^38^ However, Hek is not restricted to NMEC isolates and has been found in about one-half of urinary tract isolates, suggesting a role in urothelial barrier interaction.^39^ Indeed, in our collection of FUTI isolates, the gene *hek* was mainly present in non-K1 strains (94 non-K1 versus 3 K1 strains), most of them (*n *= 47) belonging to sequence type ST131 and O25b:H4 serotype.

TraJ is an activator of the transfer (*tra*) operon in the F plasmid that counteracts histone-like nucleoid-structuring protein (H-NS) silencing at the main transfer promoter and has been implicated in NMEC pathogenesis via specific TraJ-dependent bacterial interactions with macrophages.^40^ To our knowledge, TraJ protein has not been directly implicated in the pathogenesis of FUTI. Thus, its statistical association with FUTI in our study may be linked with a higher frequency of F-plasmids carrying other genes implicated in extra-intestinal virulence.

Although none of these five genes (*papGII*, *papC*, *hlyC*, *hek* and *traJ*) was present in more than 58% of ESBL-producing *E. coli* FUTI isolates, combining the detection of two genes (*papGII* and *traJ*) would permit the attribution of a risk-associated genetic profile (if at least one is present) to 63% of FUTI isolates versus only 24% of healthy carriage isolates. This detection can easily be performed by PCR.

The double detection of either *papGII* or *traJ* used as a screening test for high-risk ESBL-producing *E. coli* carriage has a sensitivity of 63%, a specificity of 76%, a positive predictive value of 79% and a negative predictive value of 59% (Figure S1). This means that in about one-third of cases of FUTI caused by ESBL-producing *E. coli* the carriage isolate would have been previously categorized as a ‘low-risk’ strain and the clinician incited not to use carbapenems. Thus, this low sensitivity should lead to great caution in implementing the genotyping test for the management of particularly frail patients such as young infants or the highly immunocompromised. Moreover, patients with known urologic disorder would be more likely to have a FUTI with *papGII*-negative strains.

In conclusion, ESBL-Ec strains causing FUTI in children have a genetic background similar to those found in faecal carriage; however, a few genes are not equally distributed in those two populations. Combining the detection of two genes (*papGII* and/or *traJ*) would permit the attribution of a risk-associated genetic profile to 63% of FUTI isolates versus only 24% of healthy carriage isolates. However, we believe that the empirical choice of antimicrobial in children with FUTI should result from a confrontation of clinical elements (severity of the disease, impairment of the patient) with microbiological testing such as rapid screening tests for ESBL if available and/or the proposed previous genotyping of faecal carriage isolate.

## Supplementary Material

dlac056_Supplementary_Data

## Supplementary data

Figure S1 and Tables S1 and S2 are available as Supplementary data at *JAC-AMR* Online.
