# Supplementary material for: Comparative genomic analysis of ESBL-producing Escherichia coli from faecal carriage and febrile urinary tract infection in children: a prospective multicentre study
Source: JAC Antimicrob Resist. 2022 May 21;4(3):dlac056. doi: 10.1093/jacamr/dlac056 (PMC9123598; doi:10.1093/jacamr/dlac056)
Supplement: dlac056_Supplementary_Data [file dlac056_supplementary_data.docx]

**Supplementary data**

**Table S1**. List of the putative virulence genes searched and their frequency among FUTI and carriage isolates

| **Gene(s) / allele(s)** | **FUTI isolates (n=218)** | **Fecal carriage isolates (n=154)** | **% of FUTI isolates** | **% of fecal carriage isolates** | **Chi squared p** | **Putative role** | **Accession number** |
| --- | --- | --- | --- | --- | --- | --- | --- |
| papC | 126 | 31 | 58% | 20% | 4,3092E-13 | adhesion | CU928161 (ECS88_3263) |
| papGI | 0 | 0 | 0% | 0% |  | adhesion | M20146 |
| papGI-2 | 0 | 0 | 0% | 0% |  | adhesion | AF247505 |
| papGII | 117 | 24 | 54% | 16% | 8,8079E-14 | adhesion | CU928161 (ECS88_3257) |
| papGIII | 12 | 6 | 6% | 4% | 0,476405135 | adhesion | CP000247 (ECP_4533) |
| iucC | 174 | 115 | 80% | 75% | 0,240755001 | iron uptake | CU928146 (pECS88_0011) |
| iutA | 174 | 117 | 80% | 76% | 0,376442042 | iron uptake | CU928146 (pECS88_0009) |
| fyuA | 202 | 131 | 93% | 85% | 0,018501709 | iron uptake | CU928161 (ECS88_2043) |
| irp2 | 175 | 112 | 80% | 73% | 0,087671961 | iron uptake | CU928161 (ECS88_2037) |
| iroN | 42 | 27 | 19% | 18% | 0,671783531 | iron uptake | CU928146 (pECS88_0124) |
| iroD | 45 | 29 | 21% | 19% | 0,666479064 | iron uptake | CU928146 (pECS88_0126) |
| chuA | 200 | 131 | 92% | 85% | 0,042777643 | iron uptake | CU928161 (ECS88_3913) |
| TSP-E4.C2 | 176 | 118 | 81% | 77% | 0,337422611 |  | AF222188 |
| yjaA | 148 | 102 | 68% | 66% | 0,737530267 |  | CU928161 (ECS88_4476) |
| svg | 9 | 3 | 4% | 2% | 0,241062217 |  | CU928161 (ECS88_3093) |
| cvaA | 36 | 27 | 17% | 18% | 0,796388683 | bacteriocin | CU928146 (pECS88_0117) |
| cvi | 20 | 17 | 9% | 11% | 0,553930705 | bacteriocin | CU928146 (pECS88_0114) |
| cvaC | 17 | 16 | 8% | 10% | 0,386569544 | bacteriocin | CU928146 (pECS88_0115) |
| cia | 34 | 33 | 16% | 21% | 0,149355798 | bacteriocin | CU928146 (pECS88_0104) |
| imm | 116 | 78 | 53% | 51% | 0,626145882 | bacteriocin | CU928146 (pECS88_0105) |
| shiF | 174 | 116 | 80% | 75% | 0,303298301 | iron uptake | CU928146 (pECS88_0014) |
| etsC | 21 | 18 | 10% | 12% | 0,523897326 |  | CU928146 (pECS88_0146) |
| ompTp | 22 | 20 | 10% | 13% | 0,384794886 | protectin | CU928146 (pECS88_0155) |
| hlyF | 23 | 20 | 11% | 13% | 0,469101122 | hemolysin | CU928146 (pECS88_0156) |
| mig-14 | 23 | 20 | 11% | 13% | 0,469101122 | protectin | CU928146 (pECS88_0157) |
| iss | 175 | 119 | 80% | 77% | 0,483500014 | protectin | CU928146 (pECS88_0133) |
| sitA | 196 | 125 | 90% | 81% | 0,015786142 | iron uptake | CU928146 (pECS88_0022) |
| traJ | 62 | 18 | 28% | 12% | 0,00010733 |  | CU928146 (pECS88_0064) |
| sfaS | 5 | 4 | 2% | 3% | 0,850995165 | adhesion | CP000243 (UTI89_C1114) |
| focD | 19 | 7 | 9% | 5% | 0,120239764 | adhesion | CP000243 (UTI89_C1112) |
| cnf1 | 52 | 18 | 24% | 12% | 0,003108898 | toxin | CP000243 (UTI89_C4921) |
| hek | 97 | 37 | 44% | 24% | 5,10747E-05 | invasion | CP000243 (UTI89_C4882) |
| ibeA | 6 | 0 | 3% | 0% | 0,037933172 | invasion | CP000243 (UTI89_C5035) |
| hlyC | 74 | 19 | 34% | 12% | 2,13275E-06 | hemolysin | AE014075 (c3569) |
| clbB | 27 | 12 | 12% | 8% | 0,154348885 | toxin | AE014075 (c2470) |
| clbN | 27 | 12 | 12% | 8% | 0,154348885 | toxin | AE014075 (c2455) |
| sat | 137 | 88 | 63% | 57% | 0,267933315 | toxin | AE014075 (c3619) |
| vat | 42 | 19 | 19% | 12% | 0,075460547 | toxin | AE014075 (c0393) |
| tcpC | 17 | 9 | 8% | 6% | 0,466582187 | protectin | GQ903008 |
| ihA-like | 144 | 89 | 66% | 58% | 0,104680945 | adhesion | CU928163 (ECUMN_3356) |
| mchB | 17 | 13 | 8% | 8% | 0,822389747 | bacteriocin | AE014075 (c1227) |
| mchC | 17 | 13 | 8% | 8% | 0,822389747 | bacteriocin | AJ515251 |
| mchF | 35 | 26 | 16% | 17% | 0,831746132 | bacteriocin | AF063590 |
| mcmA | 18 | 13 | 8% | 8% | 0,949386659 | bacteriocin | CP000247 |
| astA | 44 | 22 | 20% | 14% | 0,142481507 | toxin | AF161000 |
| bfpA | 0 | 0 | 0% | 0% |  | adhesion | AB247925 |
| cba | 0 | 4 | 0% | 3% | 0,016735662 | bacteriocin | FJ664727 |
| ccI | 1 | 0 | 0% | 0% | 0,40000006 | bacteriocin | AF190857 |
| cdtB | 0 | 0 | 0% | 0% |  | toxin | AJ508930 |
| celb | 1 | 3 | 0% | 2% | 0,170117678 | bacteriocin | X63621 |
| cfaC | 0 | 0 | 0% | 0% |  | adhesion | M55661 |
| cif | 0 | 0 | 0% | 0% |  | toxin | AY128537 |
| cma | 5 | 7 | 2% | 5% | 0,225982355 | bacteriocin | FJ664772 |
| cofA | 0 | 0 | 0% | 0% |  | adhesion | AB049751 |
| eatA | 11 | 4 | 5% | 3% | 0,237035662 | protectin | CP000799 |
| efa1 | 0 | 1 | 0% | 1% | 0,233502184 | adhesion | AY840983 |
| epeA | 0 | 0 | 0% | 0% |  |  | AY258503 |
| espA | 0 | 1 | 0% | 1% | 0,233502184 | adhesion | AJ225016 |
| espB | 0 | 1 | 0% | 1% | 0,233502184 | adhesion | EU871627 |
| espC | 0 | 0 | 0% | 0% |  | toxin | AF297061 |
| espF | 0 | 1 | 0% | 1% | 0,233502184 | adhesion | AF311901 |
| espI | 0 | 0 | 0% | 0% |  |  | AJ278144 |
| espJ | 0 | 0 | 0% | 0% |  | toxin | AB303060 |
| espP | 3 | 1 | 1% | 1% | 0,503207838 | protectin | HM138194 |
| etpD | 0 | 0 | 0% | 0% |  |  | AB011549 |
| f17A | 2 | 0 | 1% | 0% | 0,233324244 | adhesion | L43373 |
| f17G | 2 | 0 | 1% | 0% | 0,233324244 | adhesion | AF055312 |
| fanA | 0 | 0 | 0% | 0% |  | adhesion | X05797 |
| fasA | 0 | 0 | 0% | 0% |  | adhesion | M35257 |
| fedA | 0 | 0 | 0% | 0% |  | adhesion | M61713 |
| fedF | 0 | 0 | 0% | 0% |  | adhesion | AY970782 |
| fim41a | 0 | 0 | 0% | 0% |  | adhesion | X14354 |
| gad | 205 | 146 | 94% | 95% | 0,751752606 | protectin | CU928158 |
| ehxA | 0 | 1 | 0% | 1% | 0,233502184 | hemolysin | EF204927 |
| hlyE | 2 | 0 | 1% | 0% | 0,233324244 | hemolysin | AF052225 |
| iha | 1 | 0 | 0% | 0% | 0,40000006 | adhesion | CP000970 |
| ipaD | 0 | 0 | 0% | 0% |  | invasion | X60777 |
| ipaH9.8 | 0 | 0 | 0% | 0% |  | invasion | CP000034 |
| ireA | 39 | 14 | 18% | 9% | 0,016782279 | adhesion | CU928162 |
| K88ab | 0 | 0 | 0% | 0% |  | adhesion | CP002968 |
| katP | 0 | 0 | 0% | 0% |  | protectin | AB011549 |
| lngA | 0 | 0 | 0% | 0% |  | adhesion | AF004308 |
| lpfA | 38 | 33 | 17% | 21% | 0,333881868 | adhesion | KC207123 |
| ltcA | 0 | 1 | 0% | 1% | 0,233502184 | toxin | V00275 |
| subA | 0 | 0 | 0% | 0% |  | toxin | FJ664545 |
| saa | 0 | 0 | 0% | 0% |  | adhesion | AF399919 |
| tccP | 0 | 0 | 0% | 0% |  | adhesion | AB275121 |
| tir | 0 | 0 | 0% | 0% |  | adhesion | AF301618 |
| toxB | 0 | 0 | 0% | 0% |  | toxin | AB011549 |
| tsh | 3 | 9 | 1% | 6% | 0,016291107 | adhesion | AF218073 |
| eitA | 24 | 29 | 11% | 19% | 0,033510985 | iron uptake | NC_009837 (APECO1_O1CoBM102) |
| eitB | 22 | 24 | 10% | 16% | 0,112944115 | iron uptake | NC_009837 (APECO1_O1CoBM101) |
| virF | 0 | 0 | 0% | 0% |  |  | AF348706 |
| agg3A | 3 | 1 | 1% | 1% | 0,503207838 | adhesion | AF411067 |
| agg3B | 8 | 3 | 4% | 2% | 0,334284999 | adhesion | AF411067 |
| agg3C | 5 | 1 | 2% | 1% | 0,214992199 | adhesion | AF411067 |
| agg3D | 5 | 1 | 2% | 1% | 0,214992199 | adhesion | CU928159 |
| agg4A | 0 | 0 | 0% | 0% |  | adhesion | SSI_AA799 |
| agg4B | 0 | 0 | 0% | 0% |  | adhesion | EU637023 |
| agg4C | 0 | 0 | 0% | 0% |  | adhesion | AB255435 |
| agg4D | 0 | 0 | 0% | 0% |  | adhesion | EU637023 |
| agg5A | 2 | 0 | 1% | 0% | 0,233324244 | adhesion | SSI_AA789 |
| aggA | 6 | 0 | 3% | 0% | 0,037933172 | adhesion | AFRH01000026 |
| aggB | 6 | 0 | 3% | 0% | 0,037933172 | adhesion | U12894 |
| aggC | 6 | 0 | 3% | 0% | 0,037933172 | adhesion | U12894 |
| aggD | 6 | 0 | 3% | 0% | 0,037933172 | adhesion | U12894 |
| aggR | 10 | 3 | 5% | 2% | 0,172188811 | adhesion | AB255435 |
| capU | 25 | 15 | 11% | 10% | 0,59625072 | metabolism | CP001062 |
| eilA | 56 | 32 | 26% | 21% | 0,272494609 | adhesion | FN554766 |
| ORF3 | 10 | 3 | 5% | 2% | 0,172188811 | metabolism | FN554767 |
| ORF4 | 10 | 3 | 5% | 2% | 0,172188811 | metabolism | AB255435 |
| aafA | 0 | 2 | 0% | 1% | 0,091574665 | adhesion | AB571093 |
| aafB | 0 | 2 | 0% | 1% | 0,091574665 | adhesion | FN554767 |
| aafC | 48 | 24 | 22% | 16% | 0,121846561 | adhesion | AF114828 |
| aafD | 0 | 2 | 0% | 1% | 0,091574665 | adhesion | FN554767 |
| aaiC | 0 | 5 | 0% | 3% | 0,007395317 | adhesion | KF678353 |
| aap | 22 | 6 | 10% | 4% | 0,02568508 | adhesion | AB255435 |
| aar | 11 | 3 | 5% | 2% | 0,122017594 | adhesion | CU98159 |
| aatA | 11 | 3 | 5% | 2% | 0,122017594 | adhesion | AB255435 |
| air | 0 | 3 | 0% | 2% | 0,038534326 | adhesion | FN554766 |
| nfaE | 50 | 24 | 23% | 16% | 0,080211531 | adhesion | S61968 |
| nleA | 0 | 1 | 0% | 1% | 0,233502184 | adhesion | AM421996 |
| nleB | 0 | 1 | 0% | 1% | 0,233502184 | adhesion | CP001846 |
| nleC | 0 | 1 | 0% | 1% | 0,233502184 | adhesion | AY485823 |
| perA | 0 | 0 | 0% | 0% |  | adhesion | EF011059 |
| pet | 0 | 1 | 0% | 1% | 0,233502184 | toxin | AF056581 |
| pic | 12 | 12 | 6% | 8% | 0,376370313 | protectin | AE014075 |
| rpeA | 0 | 0 | 0% | 0% |  | adhesion | AY552473 |
| senB | 105 | 70 | 48% | 45% | 0,605918786 | toxin | CP001062 |
| sepA | 0 | 0 | 0% | 0% |  |  | Z48219 |
| sigA | 0 | 0 | 0% | 0% |  | toxin | CP000038 |
| sta1 | 0 | 0 | 0% | 0% |  | toxin | M25607 |
| stb | 0 | 0 | 0% | 0% |  | toxin | AY028790 |
| stx2A-a | 0 | 0 | 0% | 0% |  | toxin | AY633471 |
| stx2B-a | 0 | 0 | 0% | 0% |  | toxin | AY633471 |
| stx2A-b | 0 | 0 | 0% | 0% |  | toxin | AF043627 |
| stx2B-b | 0 | 0 | 0% | 0% |  | toxin | AF043627 |
| stx2A-c | 0 | 0 | 0% | 0% |  | toxin | EF441603 |
| stx2B-c | 0 | 0 | 0% | 0% |  | toxin | EF441603 |
| stx2A-d | 0 | 1 | 0% | 1% | 0,233502184 | toxin | AF479829 |
| stx2B-d | 0 | 1 | 0% | 1% | 0,233502184 | toxin | AF479829 |
| stx2A-e | 0 | 0 | 0% | 0% |  | toxin | AB252836 |
| stx2B-e | 0 | 0 | 0% | 0% |  | toxin | AB252836 |
| stx2A-f | 0 | 0 | 0% | 0% |  | toxin | AB232172 |
| stx2B-f | 0 | 0 | 0% | 0% |  | toxin | AB232172 |
| stx2A-g | 0 | 0 | 0% | 0% |  | toxin | GQ995452 |
| stx2B-g | 0 | 0 | 0% | 0% |  | toxin | GQ995452 |
| stx1A-a | 0 | 0 | 0% | 0% |  | toxin | AB083043 |
| stx1B-a | 0 | 0 | 0% | 0% |  | toxin | AB083044 |
| stx1A-c | 0 | 0 | 0% | 0% |  | toxin | AB048235 |
| stx1B-c | 0 | 0 | 0% | 0% |  | toxin | AB048235 |
| stx1A-d | 0 | 0 | 0% | 0% |  | toxin | AB050958 |
| stx1B-d | 0 | 0 | 0% | 0% |  | toxin | AB050958 |
| eae-alpha1 | 0 | 0 | 0% | 0% |  | adhesion | M58154 |
| eae-alpha2 | 0 | 0 | 0% | 0% |  | adhesion | AF530555 |
| eae-beta1 | 0 | 0 | 0% | 0% |  | adhesion | AF453441 |
| eae-beta2 | 0 | 0 | 0% | 0% |  | adhesion | AF043226 |
| eae-gamma1 | 0 | 0 | 0% | 0% |  | adhesion | AF071034 |
| eae-gamma2 | 0 | 0 | 0% | 0% |  | adhesion | AF025311 |
| eae-delta | 0 | 0 | 0% | 0% |  | adhesion | U66102 |
| eae-epsilo | 0 | 0 | 0% | 0% |  | adhesion | AF116899 |
| eae-zeta | 0 | 0 | 0% | 0% |  | adhesion | AF449417 |
| eae-eta | 0 | 0 | 0% | 0% |  | adhesion | AJ308550 |
| eae-theta | 0 | 0 | 0% | 0% |  | adhesion | AF449418 |
| eae-iota | 0 | 0 | 0% | 0% |  | adhesion | AJ308551 |
| eae-kappa | 0 | 0 | 0% | 0% |  | adhesion | AJ308552 |
| eae-lambda | 0 | 0 | 0% | 0% |  | adhesion | AF530557 |
| eae-mu | 0 | 0 | 0% | 0% |  | adhesion | KT591316 |
| eae-nu | 0 | 0 | 0% | 0% |  | adhesion | AJ705050 |
| eae-xi | 0 | 1 | 0% | 1% | 0,233502184 | adhesion | AJ705051 |
| kpsF | 190 | 122 | 87% | 79% | 0,040405763 | protectin | CU928161 (ECS88_3321) |
| kpsD | 0 | 0 | 0% | 0% |  | protectin | CU928161 (ECS88_3323) |
| K1-type neuC | 20 | 11 | 9% | 7% | 0,485022615 | protectin | CU928161 (ECS88_3328) |
| K2-type kfiA | 57 | 18 | 26% | 12% | 0,000618141 | protectin | AE014075 (c3695 CFT073) |
| K5-type KfiA | 43 | 44 | 20% | 29% | 0,047095291 | protectin | NZ_CAPM.1 (CAPMv1_690021) |
| K14-type KpsS | 8 | 9 | 4% | 6% | 0,322584264 | protectin | NC_022648 (P423_16690 JJ1886) |
| K15-type glycosyl transferase | 1 | 1 | 0% | 1% | 0,804401407 | protectin | NC_008253 (ECP_3034) |
| K52-type glycosyl transferase | 7 | 4 | 3% | 3% | 0,730761867 | protectin | CU928163 (ECUMN_3432) |
| K100-type glycosyl transferase | 18 | 14 | 8% | 9% | 0,777506283 | protectin | HG941718 (EC958_3341) |
|  |  |  |  |  |  |  |  |
| Gene/allele combinations |  |  |  |  |  |  |  |
| papGII and/or hlyC | 123 | 31 | 56% | 20% | 2,56372E-12 |  |  |
| papGII and/or hek | 139 | 53 | 64% | 34% | 2,42548E-08 |  |  |
| papGII and/or traJ | 137 | 37 | 63% | 24% | 1,46036E-13 |  |  |
| papGII and/or K2 | 125 | 35 | 57% | 23% | 3,10671E-11 |  |  |
| papGII and/or cnf1 | 123 | 31 | 56% | 20% | 2,56372E-12 |  |  |
| papGII and/or hlyC and/or hek | 139 | 53 | 64% | 34% | 2,42548E-08 |  |  |
| papGII and/or hlyC and/or traJ | 143 | 44 | 66% | 29% | 1,9965E-12 |  |  |
| papGII and/or hlyC and/or K2 | 130 | 42 | 60% | 27% | 7,01027E-10 |  |  |
| papGII and/or hlyC and/or cnf1 | 123 | 31 | 56% | 20% | 2,56372E-12 |  |  |
| papC and/or traJ | 142 | 43 | 65% | 28% | 1,53878E-12 |  |  |
| papC and/or hlyC | 128 | 32 | 59% | 21% | 3,35625E-13 |  |  |
| papC and/or hek | 141 | 53 | 65% | 34% | 8,65034E-09 |  |  |
| papC and/or cnf1 | 128 | 32 | 59% | 21% | 3,35625E-13 |  |  |
| papC and/or K2 | 133 | 42 | 61% | 27% | 1,35321E-10 |  |  |
|  |  |  |  |  |  |  |  |
| FimH types (CGE) |  |  |  |  |  |  |  |
| fimH27 | 40 | 13 | 18% | 8% | 0,007089728 |  |  |
| fimH30 | 84 | 63 | 39% | 41% | 0,644162289 |  |  |
| fimH41 | 17 | 17 | 8% | 11% | 0,285362224 |  |  |
|  |  |  |  |  |  |  |  |
| MLST (Warwick) |  |  |  |  |  |  |  |
| ST-131 | 92 | 72 | 42% | 47% | 0,38382591 |  |  |
| ST-38 | 23 | 9 | 11% | 6% | 0,110823287 |  |  |
| ST-69 | 15 | 6 | 7% | 4% | 0,219246364 |  |  |
| ST-73 | 9 | 4 | 4% | 3% | 0,428358656 |  |  |
| ST-95 | 9 | 3 | 4% | 2% | 0,241062217 |  |  |
| phylogroup B2 | 140 | 92 | 64% | 60% | 0,37969337 |  |  |
|  |  |  |  |  |  |  |  |
| Serotype (CGE) |  |  |  |  |  |  |  |
| O16:H5 | 17 | 14 | 8% | 9% | 0,656797418 |  |  |
| O25:H4 | 80 | 61 | 37% | 40% | 0,568382691 |  |  |
| O6:H1 | 6 | 3 | 3% | 2% | 0,619014972 |  |  |
| O75:H5 | 7 | 3 | 3% | 2% | 0,458200649 |  |  |
| O86:H18 | 11 | 9 | 5% | 6% | 0,736700428 |  |  |

**Table S2:** Quality of the sequencing data

| **origin** | **isolate** | **genome_size (bp)** | **contigs** | **n50** | **coverage** | **mlst** | **mlst_genes** | **ESBL gene** | **fimH** |
| --- | --- | --- | --- | --- | --- | --- | --- | --- | --- |
| urine | ABE12 | 5295550 | 339 | 141512 | 52 | ecoli[Unknown ST] | ecoli[ADK-334,FUMC-40,GYRB-25,ICD-13,MDH-36,PURA-28,RECA-29] | blaCTX-M-15 | 30 |
| urine | ABE3 | 5198229 | 254 | 192296 | 117 | ecoli[ST-354] | ecoli[ADK-85,FUMC-88,GYRB-78,ICD-29,MDH-59,PURA-58,RECA-62] | blaCTX-M-15 | ND |
| urine | ABE6 | 5288076 | 226 | 191429 | 145 | ecoli[ST-131] | ecoli[ADK-53,FUMC-40,GYRB-47,ICD-13,MDH-36,PURA-28,RECA-29] | blaCTX-M-15 | 30 |
| urine | ABE8 | 5250937 | 217 | 190772 | 113 | ecoli[ST-131] | ecoli[ADK-53,FUMC-40,GYRB-47,ICD-13,MDH-36,PURA-28,RECA-29] | blaCTX-M-15 | 30 |
| urine | ABE9 | 4724858 | 95 | 337109 | 94 | ecoli[ST-569] | ecoli[ADK-13,FUMC-38,GYRB-84,ICD-13,MDH-17,PURA-64,RECA-34] | blaCTX-M-1 | 5 |
| urine | AMI1 | 5184943 | 267 | 190765 | 116 | ecoli[ST-131] | ecoli[ADK-53,FUMC-40,GYRB-47,ICD-13,MDH-36,PURA-28,RECA-29] | blaCTX-M-15 | 30 |
| urine | AMI10 | 5300218 | 311 | 214124 | 82 | ecoli[ST-73] | ecoli[ADK-36,FUMC-24,GYRB-9,ICD-13,MDH-17,PURA-11,RECA-25] | blaCTX-M-15 | 10 |
| urine | AMI14 | 5155332 | 203 | 170373 | 82 | ecoli[ST-131] | ecoli[ADK-53,FUMC-40,GYRB-47,ICD-13,MDH-36,PURA-28,RECA-29] | blaCTX-M-15 | 30 |
| urine | AMI15 | 5105662 | 208 | 182314 | 89 | ecoli[ST-131] | ecoli[ADK-53,FUMC-40,GYRB-47,ICD-13,MDH-36,PURA-28,RECA-29] | blaCTX-M-27 | 30 |
| urine | AMI16 | 5346688 | 439 | 145189 | 140 | ecoli[ST-117] | ecoli[ADK-20,FUMC-45,GYRB-41,ICD-43,MDH-5,PURA-32,RECA-2] | blaCTX-M-14 | 97 |
| urine | AMI18 | 5407604 | 368 | 154831 | 64 | ecoli[ST-38] | ecoli[ADK-4,FUMC-26,GYRB-2,ICD-25,MDH-5,PURA-5,RECA-19] | blaCTX-M-14 | 5 |
| urine | AMI2 | 5133246 | 186 | 175122 | 61 | ecoli[ST-1982] | ecoli[ADK-53,FUMC-40,GYRB-254,ICD-13,MDH-36,PURA-28,RECA-29] | blaCTX-M-27 | 30 |
| urine | AMI3 | 5275376 | 238 | 191445 | 78 | ecoli[ST-131] | ecoli[ADK-53,FUMC-40,GYRB-47,ICD-13,MDH-36,PURA-28,RECA-29] | blaCTX-M-15 | 30 |
| urine | AMI4 | 5446644 | 376 | 259133 | 71 | ecoli[ST-69] | ecoli[ADK-21,FUMC-35,GYRB-27,ICD-6,MDH-5,PURA-5,RECA-4] | blaCTX-M-14 | 27 |
| urine | AMI5 | 5344791 | 273 | 173842 | 89 | ecoli[ST-131] | ecoli[ADK-53,FUMC-40,GYRB-47,ICD-13,MDH-36,PURA-28,RECA-29] | blaCTX-M-15 | 30 |
| urine | AMI7 | 5116875 | 200 | 230020 | 97 | ecoli[ST-131] | ecoli[ADK-53,FUMC-40,GYRB-47,ICD-13,MDH-36,PURA-28,RECA-29] | blaCTX-M-15 | 41 |
| urine | AMI8 | 5320967 | 244 | 186885 | 83 | ecoli[ST-131] | ecoli[ADK-53,FUMC-40,GYRB-47,ICD-13,MDH-36,PURA-28,RECA-29] | blaCTX-M-15 | 30 |
| urine | AMI9 | 5213276 | 247 | 135287 | 96 | ecoli[ST-69] | ecoli[ADK-21,FUMC-35,GYRB-27,ICD-6,MDH-5,PURA-5,RECA-4] | blaCTX-M-15 | 27 |
| urine | APE2 | 5416205 | 330 | 233566 | 196 | ecoli[ST-14] | ecoli[ADK-14,FUMC-14,GYRB-10,ICD-14,MDH-17,PURA-7,RECA-10] | blaCTX-M-15 | 27 |
| urine | APE4 | 5354492 | 264 | 147769 | 104 | ecoli[ST-131] | ecoli[ADK-53,FUMC-40,GYRB-47,ICD-13,MDH-36,PURA-28,RECA-29] | blaCTX-M-14 | 30 |
| urine | APE6 | 5354583 | 514 | 132188 | 105 | ecoli[ST-38] | ecoli[ADK-4,FUMC-26,GYRB-2,ICD-25,MDH-5,PURA-5,RECA-19] | blaCTX-M-15 | 5 |
| urine | APE8 | 5281631 | 246 | 189125 | 76 | ecoli[ST-131] | ecoli[ADK-53,FUMC-40,GYRB-47,ICD-13,MDH-36,PURA-28,RECA-29] | blaCTX-M-15 | 30 |
| urine | ATR1 | 5462685 | 354 | 136867 | 57 | ecoli[ST-69] | ecoli[ADK-21,FUMC-35,GYRB-27,ICD-6,MDH-5,PURA-5,RECA-4] | blaCTX-M-27 | 27 |
| urine | ATR11 | 5083058 | 274 | 159837 | 90 | ecoli[ST-131] | ecoli[ADK-53,FUMC-40,GYRB-47,ICD-13,MDH-36,PURA-28,RECA-29] | blaCTX-M-27 | 30 |
| urine | ATR13 | 5549148 | 344 | 105607 | 92 | ecoli[ST-57] | ecoli[ADK-6,FUMC-31,GYRB-5,ICD-28,MDH-1,PURA-1,RECA-2] | blaSHV-12 | 27 |
| urine | ATR14 | 5312334 | 300 | 189554 | 99 | ecoli[ST-131] | ecoli[ADK-53,FUMC-40,GYRB-47,ICD-13,MDH-36,PURA-28,RECA-29] | blaCTX-M-15 | 30 |
| urine | ATR18 | 5359087 | 267 | 163733 | 117 | ecoli[ST-131] | ecoli[ADK-53,FUMC-40,GYRB-47,ICD-13,MDH-36,PURA-28,RECA-29] | blaCTX-M-15 | 30 |
| urine | ATR2 | 5558048 | 532 | 139213 | 110 | ecoli[ST-38] | ecoli[ADK-4,FUMC-26,GYRB-2,ICD-25,MDH-5,PURA-5,RECA-19] | blaCTX-M-3 | 65 |
| urine | ATR20 | 5309210 | 360 | 172988 | 55 | ecoli[ST-131] | ecoli[ADK-53,FUMC-40,GYRB-47,ICD-13,MDH-36,PURA-28,RECA-29] | blaCTX-M-15 | 30 |
| urine | ATR21 | 4840200 | 174 | 124287 | 121 | ecoli[ST-453] | ecoli[ADK-99,FUMC-6,GYRB-33,ICD-33,MDH-24,PURA-8,RECA-7] | blaCTX-M-15 | 31 |
| urine | ATR22 | 5245604 | 352 | 97339 | 117 | ecoli[ST-69] | ecoli[ADK-21,FUMC-35,GYRB-27,ICD-6,MDH-5,PURA-5,RECA-4] | blaCTX-M-14 | 27 |
| urine | ATR23 | 5435567 | 419 | 148854 | 100 | ecoli[ST-38] | ecoli[ADK-4,FUMC-26,GYRB-2,ICD-25,MDH-5,PURA-5,RECA-19] | blaCTX-M-14 | 5 |
| urine | ATR24 | 5307541 | 288 | 200970 | 118 | ecoli[ST-69] | ecoli[ADK-21,FUMC-35,GYRB-27,ICD-6,MDH-5,PURA-5,RECA-4] | blaCTX-M-15 | 27 |
| urine | ATR25 | 5048284 | 152 | 322941 | 154 | ecoli[ST-1193] | ecoli[ADK-14,FUMC-14,GYRB-10,ICD-200,MDH-17,PURA-7,RECA-10] | blaCTX-M-27 | 64 |
| urine | ATR28 | 5382264 | 339 | 241766 | 162 | ecoli[ST-12] | ecoli[ADK-13,FUMC-13,GYRB-9,ICD-13,MDH-16,PURA-10,RECA-9] | blaCTX-M-15 | 106 |
| urine | ATR29 | 5090710 | 206 | 169689 | 84 | ecoli[ST-131] | ecoli[ADK-53,FUMC-40,GYRB-47,ICD-13,MDH-36,PURA-28,RECA-29] | blaCTX-M-27 | 30 |
| urine | ATR3 | 5412642 | 198 | 175438 | 79 | ecoli[ST-131] | ecoli[ADK-53,FUMC-40,GYRB-47,ICD-13,MDH-36,PURA-28,RECA-29] | blaCTX-M-15 | 30 |
| urine | ATR33 | 5203070 | 172 | 237227 | 267 | ecoli[ST-1193] | ecoli[ADK-14,FUMC-14,GYRB-10,ICD-200,MDH-17,PURA-7,RECA-10] | blaCTX-M-15 | 64 |
| urine | ATR34 | 5535658 | 438 | 189382 | 93 | ecoli[ST-131] | ecoli[ADK-53,FUMC-40,GYRB-47,ICD-13,MDH-36,PURA-28,RECA-29] | blaCTX-M-15 | 30 |
| urine | ATR35 | 5416484 | 279 | 182937 | 97 | ecoli[ST-131] | ecoli[ADK-53,FUMC-40,GYRB-47,ICD-13,MDH-36,PURA-28,RECA-29] | blaCTX-M-15 | 30 |
| urine | ATR38 | 5285411 | 278 | 180706 | 89 | ecoli[ST-69] | ecoli[ADK-21,FUMC-35,GYRB-27,ICD-6,MDH-5,PURA-5,RECA-4] | blaCTX-M-32 | 27 |
| urine | ATR39 | 5373146 | 257 | 180917 | 80 | ecoli[ST-131] | ecoli[ADK-53,FUMC-40,GYRB-47,ICD-13,MDH-36,PURA-28,RECA-29] | blaCTX-M-15 | 30 |
| urine | ATR4 | 5197832 | 245 | 170571 | 123 | ecoli[ST-131] | ecoli[ADK-53,FUMC-40,GYRB-47,ICD-13,MDH-36,PURA-28,RECA-29] | blaCTX-M-15 | 30 |
| urine | ATR40 | 5407036 | 308 | 202150 | 76 | ecoli[ST-131] | ecoli[ADK-53,FUMC-40,GYRB-47,ICD-13,MDH-36,PURA-28,RECA-29] | blaCTX-M-15 | 30 |
| urine | ATR42 | 5232832 | 365 | 188621 | 97 | ecoli[ST-95] | ecoli[ADK-37,FUMC-38,GYRB-19,ICD-37,MDH-17,PURA-11,RECA-26] | blaCTX-M-15 | 27 |
| urine | ATR46 | 5040169 | 219 | 170414 | 82 | ecoli[ST-131] | ecoli[ADK-53,FUMC-40,GYRB-47,ICD-13,MDH-36,PURA-28,RECA-29] | blaCTX-M-27 | 30 |
| urine | ATR5 | 5261601 | 210 | 192418 | 93 | ecoli[ST-131] | ecoli[ADK-53,FUMC-40,GYRB-47,ICD-13,MDH-36,PURA-28,RECA-29] | blaCTX-M-15 | 30 |
| urine | ATR6 | 5101347 | 216 | 157348 | 105 | ecoli[ST-131] | ecoli[ADK-53,FUMC-40,GYRB-47,ICD-13,MDH-36,PURA-28,RECA-29] | blaCTX-M-14 | 30 |
| urine | ATR7 | 5291438 | 226 | 189106 | 118 | ecoli[ST-131] | ecoli[ADK-53,FUMC-40,GYRB-47,ICD-13,MDH-36,PURA-28,RECA-29] | blaCTX-M-15 | 30 |
| urine | BIC1 | 5357125 | 283 | 195845 | 609 | ecoli[ST-69] | ecoli[ADK-21,FUMC-35,GYRB-27,ICD-6,MDH-5,PURA-5,RECA-4] | blaCTX-M-14 | 27 |
| urine | BIC10 | 5077703 | 290 | 199744 | 117 | ecoli[ST-131] | ecoli[ADK-53,FUMC-40,GYRB-47,ICD-13,MDH-36,PURA-28,RECA-29] | blaCTX-M-27 | 41 |
| urine | BIC12 | 5241705 | 259 | 177006 | 110 | ecoli[ST-131] | ecoli[ADK-53,FUMC-40,GYRB-47,ICD-13,MDH-36,PURA-28,RECA-29] | blaCTX-M-15 | 30 |
| urine | BIC13 | 5250616 | 254 | 160044 | 158 | ecoli[ST-131] | ecoli[ADK-53,FUMC-40,GYRB-47,ICD-13,MDH-36,PURA-28,RECA-29] | blaCTX-M-15 | 30 |
| urine | BIC14 | 5374428 | 413 | 106380 | 99 | ecoli[ST-405] | ecoli[ADK-35,FUMC-37,GYRB-29,ICD-25,MDH-4,PURA-5,RECA-73] | blaCTX-M-14 | 27 |
| urine | BIC16 | 5150016 | 602 | 67297 | 95 | ecoli[ST-1312] | ecoli[ADK-6,FUMC-11,GYRB-4,ICD-8,MDH-8,PURA-78,RECA-2] | blaCTX-M-15 | 198 |
| urine | BIC17 | 5136969 | 201 | 191462 | 154 | ecoli[ST-131] | ecoli[ADK-53,FUMC-40,GYRB-47,ICD-13,MDH-36,PURA-28,RECA-29] | blaCTX-M-14 | 41 |
| urine | BIC19 | 5426070 | 391 | 153105 | 81 | ecoli[ST-73] | ecoli[ADK-36,FUMC-24,GYRB-9,ICD-13,MDH-17,PURA-11,RECA-25] | blaCTX-M-3 | 10 |
| urine | BIC22 | 5091606 | 261 | 237079 | 76 | ecoli[ST-1193] | ecoli[ADK-14,FUMC-14,GYRB-10,ICD-200,MDH-17,PURA-7,RECA-10] | blaCTX-M-15 | 64 |
| urine | BIC24 | 5141571 | 235 | 191473 | 91 | ecoli[ST-131] | ecoli[ADK-53,FUMC-40,GYRB-47,ICD-13,MDH-36,PURA-28,RECA-29] | blaCTX-M-27 | 234 |
| urine | BIC25 | 5203629 | 230 | 159235 | 79 | ecoli[ST-131] | ecoli[ADK-53,FUMC-40,GYRB-47,ICD-13,MDH-36,PURA-28,RECA-29] | blaCTX-M-15 | 30 |
| urine | BIC26 | 5342658 | 272 | 189991 | 80 | ecoli[ST-131] | ecoli[ADK-53,FUMC-40,GYRB-47,ICD-13,MDH-36,PURA-28,RECA-29] | blaCTX-M-15 | 30 |
| urine | BIC27 | 5244779 | 215 | 284312 | 94 | ecoli[ST-131] | ecoli[ADK-53,FUMC-40,GYRB-47,ICD-13,MDH-36,PURA-28,RECA-29] | blaCTX-M-14 | 30 |
| urine | BIC28 | 5445112 | 542 | 145448 | 102 | ecoli[ST-38] | ecoli[ADK-4,FUMC-26,GYRB-2,ICD-25,MDH-5,PURA-5,RECA-19] | blaCTX-M-27 | ND |
| urine | BIC29 | 5084907 | 173 | 202031 | 78 | ecoli[ST-131] | ecoli[ADK-53,FUMC-40,GYRB-47,ICD-13,MDH-36,PURA-28,RECA-29] | blaCTX-M-14 | 30 |
| urine | BIC4 | 5362940 | 213 | 180854 | 80 | ecoli[ST-131] | ecoli[ADK-53,FUMC-40,GYRB-47,ICD-13,MDH-36,PURA-28,RECA-29] | blaCTX-M-15 | 30 |
| urine | BIC6 | 5363175 | 460 | 204764 | 82 | ecoli[ST-131] | ecoli[ADK-53,FUMC-40,GYRB-47,ICD-13,MDH-36,PURA-28,RECA-29] | blaCTX-M-15 | 30 |
| urine | BIC8 | 5416573 | 584 | 98250 | 63 | ecoli[ST-38] | ecoli[ADK-4,FUMC-26,GYRB-2,ICD-25,MDH-5,PURA-5,RECA-19] | blaCTX-M-27 | ND |
| urine | BIC9 | 4806923 | 188 | 156801 | 113 | ecoli[ST-540] | ecoli[ADK-6,FUMC-7,GYRB-57,ICD-1,MDH-8,PURA-8,RECA-2] | blaCTX-M-32 | 54 |
| urine | CHI1 | 5355472 | 563 | 143248 | 110 | ecoli[ST-73] | ecoli[ADK-36,FUMC-24,GYRB-9,ICD-13,MDH-17,PURA-11,RECA-25] | blaCTX-M-14 | 30 |
| urine | CHI11 | 5128729 | 155 | 221414 | 88 | ecoli[ST-1722] | ecoli[ADK-101,FUMC-4,GYRB-97,ICD-29,MDH-70,PURA-158,RECA-2] | blaCTX-M-27 | 153 |
| urine | CHI12 | 5430500 | 382 | 108855 | 91 | ecoli[ST-62] | ecoli[ADK-28,FUMC-33,GYRB-25,ICD-29,MDH-7,PURA-11,RECA-24] | blaCTX-M-15 | 44 |
| urine | CHI13 | 5230230 | 262 | 182811 | 88 | ecoli[ST-131] | ecoli[ADK-53,FUMC-40,GYRB-47,ICD-13,MDH-36,PURA-28,RECA-29] | blaCTX-M-15 | 30 |
| urine | CHI15 | 5207911 | 145 | 290044 | 88 | ecoli[ST-95] | ecoli[ADK-37,FUMC-38,GYRB-19,ICD-37,MDH-17,PURA-11,RECA-26] | blaCTX-M-15 | 41 |
| urine | CHI16 | 5289240 | 294 | 202098 | 242 | ecoli[ST-73] | ecoli[ADK-36,FUMC-24,GYRB-9,ICD-13,MDH-17,PURA-11,RECA-25] | blaCTX-M-15 | 184 |
| urine | CHI17 | 5339918 | 276 | 173686 | 162 | ecoli[ST-131] | ecoli[ADK-53,FUMC-40,GYRB-47,ICD-13,MDH-36,PURA-28,RECA-29] | blaCTX-M-15 | 30 |
| urine | CHI2 | 5394236 | 319 | 186743 | 98 | ecoli[ST-69] | ecoli[ADK-21,FUMC-35,GYRB-27,ICD-6,MDH-5,PURA-5,RECA-4] | blaCTX-M-14 | 27 |
| urine | CHI20 | 5394387 | 963 | 57464 | 87 | ecoli[ST-38] | ecoli[ADK-4,FUMC-26,GYRB-2,ICD-25,MDH-5,PURA-5,RECA-19] | blaCTX-M-27 | ND |
| urine | CHI22 | 5154379 | 311 | 177321 | 102 | ecoli[ST-73] | ecoli[ADK-36,FUMC-24,GYRB-9,ICD-13,MDH-17,PURA-11,RECA-25] | blaCTX-M-14 | 103 |
| urine | CHI23 | 5222594 | 342 | 205394 | 67 | ecoli[ST-117] | ecoli[ADK-20,FUMC-45,GYRB-41,ICD-43,MDH-5,PURA-32,RECA-2] | blaCTX-M-1 | 97 |
| urine | CHI24 | 4916424 | 235 | 93405 | 105 | ecoli[ST-1421] | ecoli[ADK-8,FUMC-7,GYRB-1,ICD-8,MDH-8,PURA-8,RECA-2] | blaCTX-M-1 | 34 |
| urine | CHI26 | 5146245 | 196 | 222825 | 255 | ecoli[ST-131] | ecoli[ADK-53,FUMC-40,GYRB-47,ICD-13,MDH-36,PURA-28,RECA-29] | blaCTX-M-15 | 30 |
| urine | CHI27 | 5505792 | 597 | 114269 | 85 | ecoli[ST-38] | ecoli[ADK-4,FUMC-26,GYRB-2,ICD-25,MDH-5,PURA-5,RECA-19] | blaCTX-M-27 | ND |
| urine | CHI28 | 5332674 | 234 | 159055 | 87 | ecoli[ST-131] | ecoli[ADK-53,FUMC-40,GYRB-47,ICD-13,MDH-36,PURA-28,RECA-29] | blaCTX-M-15 | 30 |
| urine | CHI29 | 5178220 | 270 | 118918 | 222 | ecoli[ST-10] | ecoli[ADK-10,FUMC-11,GYRB-4,ICD-8,MDH-8,PURA-8,RECA-2] | blaCTX-M-15 | 28 |
| urine | CHI3 | 5202385 | 252 | 258103 | 85 | ecoli[ST-14] | ecoli[ADK-14,FUMC-14,GYRB-10,ICD-14,MDH-17,PURA-7,RECA-10] | blaSHV-12 | 27 |
| urine | CHI30 | 5335458 | 501 | 130318 | 39 | ecoli[ST-4363] | ecoli[ADK-13,FUMC-13,GYRB-9,ICD-13,MDH-16,PURA-5,RECA-9] | blaCTX-M-15 | 27 |
| urine | CHI33 | 5288760 | 382 | 163018 | 99 | ecoli[ST-131] | ecoli[ADK-53,FUMC-40,GYRB-47,ICD-13,MDH-36,PURA-28,RECA-29] | blaCTX-M-15 | 30 |
| urine | CHI35 | 5264290 | 205 | 161469 | 84 | ecoli[ST-131] | ecoli[ADK-53,FUMC-40,GYRB-47,ICD-13,MDH-36,PURA-28,RECA-29] | blaCTX-M-15 | 30 |
| urine | CHI4 | 5200619 | 285 | 105643 | 78 | ecoli[ST-10] | ecoli[ADK-10,FUMC-11,GYRB-4,ICD-8,MDH-8,PURA-8,RECA-2] | blaCTX-M-15 | 94 |
| urine | CHI5 | 5057928 | 159 | 191429 | 126 | ecoli[ST-131] | ecoli[ADK-53,FUMC-40,GYRB-47,ICD-13,MDH-36,PURA-28,RECA-29] | blaCTX-M-27 | 30 |
| urine | CHI6 | 5073218 | 235 | 168339 | 95 | ecoli[ST-636] | ecoli[ADK-13,FUMC-108,GYRB-10,ICD-97,MDH-18,PURA-68,RECA-93] | blaCTX-M-15 | ND |
| urine | CHI8 | 4822877 | 73 | 314152 | 103 | ecoli[ST-538] | ecoli[ADK-13,FUMC-40,GYRB-19,ICD-13,MDH-36,PURA-28,RECA-30] | blaSHV-12 | 20 |
| urine | CHI9 | 5406409 | 299 | 147865 | 94 | ecoli[ST-38] | ecoli[ADK-4,FUMC-26,GYRB-2,ICD-25,MDH-5,PURA-5,RECA-19] | blaCTX-M-14 | 5 |
| urine | CHO1 | 5128690 | 317 | 255412 | 111 | ecoli[ST-88] | ecoli[ADK-6,FUMC-4,GYRB-12,ICD-1,MDH-20,PURA-12,RECA-7] | blaCTX-M-1 | 54 |
| urine | CHO2 | 5506704 | 782 | 88871 | 96 | ecoli[ST-131] | ecoli[ADK-53,FUMC-40,GYRB-47,ICD-13,MDH-36,PURA-28,RECA-29] | blaCTX-M-15 | 30 |
| urine | DOU1 | 5263767 | 268 | 142666 | 91 | ecoli[ST-405] | ecoli[ADK-35,FUMC-37,GYRB-29,ICD-25,MDH-4,PURA-5,RECA-73] | blaCTX-M-14 | 27 |
| urine | JVE12 | 5287833 | 348 | 126088 | 100 | ecoli[ST-405] | ecoli[ADK-35,FUMC-37,GYRB-29,ICD-25,MDH-4,PURA-5,RECA-73] | blaCTX-M-14 | 27 |
| urine | JVE13 | 5109709 | 193 | 162884 | 95 | ecoli[ST-131] | ecoli[ADK-53,FUMC-40,GYRB-47,ICD-13,MDH-36,PURA-28,RECA-29] | blaCTX-M-15 | 30 |
| urine | JVE14 | 5179505 | 256 | 231279 | 83 | ecoli[ST-73] | ecoli[ADK-36,FUMC-24,GYRB-9,ICD-13,MDH-17,PURA-11,RECA-25] | blaCTX-M-15 | 10 |
| urine | JVE19 | 5354838 | 364 | 164825 | 79 | ecoli[ST-131] | ecoli[ADK-53,FUMC-40,GYRB-47,ICD-13,MDH-36,PURA-28,RECA-29] | blaCTX-M-15 | 41 |
| urine | JVE22 | 5479693 | 437 | 130366 | 103 | ecoli[ST-38] | ecoli[ADK-4,FUMC-26,GYRB-2,ICD-25,MDH-5,PURA-5,RECA-19] | blaCTX-M-27 | ND |
| urine | JVE24 | 5223756 | 209 | 191640 | 116 | ecoli[ST-95] | ecoli[ADK-37,FUMC-38,GYRB-19,ICD-37,MDH-17,PURA-11,RECA-26] | blaCTX-M-15 | 27 |
| urine | JVE25 | 5320655 | 228 | 170571 | 89 | ecoli[ST-131] | ecoli[ADK-53,FUMC-40,GYRB-47,ICD-13,MDH-36,PURA-28,RECA-29] | blaCTX-M-15 | 30 |
| urine | JVE26 | 5424167 | 457 | 103704 | 95 | ecoli[ST-2020] | ecoli[ADK-21,FUMC-35,GYRB-27,ICD-6,MDH-220,PURA-5,RECA-4] | blaCTX-M-15 | 27 |
| urine | JVE27 | 5489266 | 551 | 164618 | 76 | ecoli[ST-69] | ecoli[ADK-21,FUMC-35,GYRB-27,ICD-6,MDH-5,PURA-5,RECA-4] | blaCTX-M-14 | 27 |
| urine | JVE28 | 5318848 | 273 | 228825 | 78 | ecoli[ST-131] | ecoli[ADK-53,FUMC-40,GYRB-47,ICD-13,MDH-36,PURA-28,RECA-29] | blaCTX-M-15 | 30 |
| urine | JVE29 | 5077842 | 147 | 225579 | 95 | ecoli[ST-354] | ecoli[ADK-85,FUMC-88,GYRB-78,ICD-29,MDH-59,PURA-58,RECA-62] | blaCTX-M-24 | 58 |
| urine | JVE3 | 5005496 | 200 | 239832 | 119 | ecoli[ST-95] | ecoli[ADK-37,FUMC-38,GYRB-19,ICD-37,MDH-17,PURA-11,RECA-26] | blaCTX-M-15 | 27 |
| urine | JVE4 | 4990597 | 187 | 218503 | 82 | ecoli[ST-131] | ecoli[ADK-53,FUMC-40,GYRB-47,ICD-13,MDH-36,PURA-28,RECA-29] | blaCTX-M-14 | 41 |
| urine | JVE7 | 5241417 | 255 | 230804 | 148 | ecoli[ST-69] | ecoli[ADK-21,FUMC-35,GYRB-27,ICD-6,MDH-5,PURA-5,RECA-4] | blaCTX-M-27 | 483 |
| urine | LMR2 | 5542701 | 346 | 164560 | 79 | ecoli[ST-648] | ecoli[ADK-92,FUMC-4,GYRB-87,ICD-96,MDH-70,PURA-58,RECA-2] | blaCTX-M-15 | ND |
| urine | LYO1 | 5340053 | 247 | 155975 | 149 | ecoli[ST-38] | ecoli[ADK-4,FUMC-26,GYRB-2,ICD-25,MDH-5,PURA-5,RECA-19] | blaCTX-M-15 | ND |
| urine | LYO10 | 4889580 | 187 | 257965 | 99 | ecoli[ST-2599] | ecoli[ADK-267,FUMC-6,GYRB-5,ICD-26,MDH-9,PURA-13,RECA-98] | blaCTX-M-14 | 32 |
| urine | LYO13 | 5307709 | 240 | 260135 | 93 | ecoli[ST-12] | ecoli[ADK-13,FUMC-13,GYRB-9,ICD-13,MDH-16,PURA-10,RECA-9] | blaCTX-M-15 | 27 |
| urine | LYO15 | 5385426 | 429 | 191468 | 82 | ecoli[ST-131] | ecoli[ADK-53,FUMC-40,GYRB-47,ICD-13,MDH-36,PURA-28,RECA-29] | blaCTX-M-15 | 41 |
| urine | LYO21 | 5438878 | 717 | 60312 | 53 | ecoli[Unknown ST] | ecoli[ADK-21,FUMC-35,GYRB-27,ICD-6,MDH-5,PURA-5,RECA-118] | blaCTX-M-15 | 27 |
| urine | LYO23 | 5118896 | 218 | 215544 | 193 | ecoli[ST-131] | ecoli[ADK-53,FUMC-40,GYRB-47,ICD-13,MDH-36,PURA-28,RECA-29] | blaCTX-M-15 | 41 |
| urine | LYO25 | 5464027 | 332 | 114849 | 113 | ecoli[ST-405] | ecoli[ADK-35,FUMC-37,GYRB-29,ICD-25,MDH-4,PURA-5,RECA-73] | blaCTX-M-14 | 27 |
| urine | LYO26 | 5185067 | 162 | 191781 | 110 | ecoli[ST-131] | ecoli[ADK-53,FUMC-40,GYRB-47,ICD-13,MDH-36,PURA-28,RECA-29] | blaCTX-M-15 | 30 |
| urine | LYO27 | 5418944 | 266 | 180887 | 89 | ecoli[ST-131] | ecoli[ADK-53,FUMC-40,GYRB-47,ICD-13,MDH-36,PURA-28,RECA-29] | blaCTX-M-15 | 30 |
| urine | LYO29 | 5259485 | 241 | 212057 | 111 | ecoli[ST-357] | ecoli[ADK-13,FUMC-40,GYRB-13,ICD-13,MDH-23,PURA-25,RECA-66] | blaCTX-M-1 | 21 |
| urine | LYO3 | 5142419 | 201 | 292635 | 94 | ecoli[ST-6355] | ecoli[ADK-13,FUMC-13,GYRB-9,ICD-13,MDH-16,PURA-10,RECA-449] | blaCTX-M-15 | 106 |
| urine | LYO30 | 5233968 | 235 | 182739 | 108 | ecoli[ST-69] | ecoli[ADK-21,FUMC-35,GYRB-27,ICD-6,MDH-5,PURA-5,RECA-4] | blaCTX-M-14 | 27 |
| urine | LYO34 | 5037867 | 676 | 140960 | 115 | ecoli[ST-10] | ecoli[ADK-10,FUMC-11,GYRB-4,ICD-8,MDH-8,PURA-8,RECA-2] | blaCTX-M-32 | 54 |
| urine | LYO36 | 5691775 | 722 | 93094 | 134 | ecoli[ST-405] | ecoli[ADK-35,FUMC-37,GYRB-29,ICD-25,MDH-4,PURA-5,RECA-73] | blaCTX-M-14 | 27 |
| urine | LYO38 | 5366309 | 353 | 176004 | 81 | ecoli[ST-131] | ecoli[ADK-53,FUMC-40,GYRB-47,ICD-13,MDH-36,PURA-28,RECA-29] | blaCTX-M-15 | 30 |
| urine | LYO4 | 5127543 | 90 | 360156 | 141 | ecoli[ST-998] | ecoli[ADK-13,FUMC-52,GYRB-156,ICD-14,MDH-17,PURA-25,RECA-17] | blaCTX-M-14 | 76 |
| urine | LYO41 | 5305862 | 432 | 106816 | 41 | ecoli[ST-38] | ecoli[ADK-4,FUMC-26,GYRB-2,ICD-25,MDH-5,PURA-5,RECA-19] | blaCTX-M-15 | 302 |
| urine | LYO7 | 5339009 | 244 | 191421 | 146 | ecoli[ST-131] | ecoli[ADK-53,FUMC-40,GYRB-47,ICD-13,MDH-36,PURA-28,RECA-29] | blaCTX-M-15 | 30 |
| urine | MJT1 | 5032377 | 191 | 159062 | 84 | ecoli[ST-131] | ecoli[ADK-53,FUMC-40,GYRB-47,ICD-13,MDH-36,PURA-28,RECA-29] | blaCTX-M-27 | 30 |
| urine | MJT2 | 5164496 | 226 | 235790 | 88 | ecoli[ST-12] | ecoli[ADK-13,FUMC-13,GYRB-9,ICD-13,MDH-16,PURA-10,RECA-9] | blaCTX-M-55 | 106 |
| urine | NAN8 | 5284176 | 245 | 159096 | 84 | ecoli[ST-131] | ecoli[ADK-53,FUMC-40,GYRB-47,ICD-13,MDH-36,PURA-28,RECA-29] | blaCTX-M-27 | 30 |
| urine | NCK1 | 5197738 | 332 | 208032 | 99 | ecoli[ST-5041] | ecoli[ADK-21,FUMC-35,GYRB-423,ICD-52,MDH-5,PURA-5,RECA-4] | blaCTX-M-15 | 30 |
| urine | NCK10 | 5415352 | 381 | 154829 | 115 | ecoli[ST-38] | ecoli[ADK-4,FUMC-26,GYRB-2,ICD-25,MDH-5,PURA-5,RECA-19] | blaCTX-M-14 | 5 |
| urine | NCK15 | 5243422 | 199 | 183987 | 64 | ecoli[ST-131] | ecoli[ADK-53,FUMC-40,GYRB-47,ICD-13,MDH-36,PURA-28,RECA-29] | blaCTX-M-15 | 30 |
| urine | NCK17 | 5403509 | 255 | 191474 | 64 | ecoli[ST-131] | ecoli[ADK-53,FUMC-40,GYRB-47,ICD-13,MDH-36,PURA-28,RECA-29] | blaCTX-M-15 | 30 |
| urine | NCK2 | 5269947 | 230 | 181518 | 91 | ecoli[ST-131] | ecoli[ADK-53,FUMC-40,GYRB-47,ICD-13,MDH-36,PURA-28,RECA-29] | blaCTX-M-15 | 30 |
| urine | NCK20 | 5103976 | 237 | 285884 | 417 | ecoli[ST-636] | ecoli[ADK-13,FUMC-108,GYRB-10,ICD-97,MDH-18,PURA-68,RECA-93] | blaSHV-12 | ND |
| urine | NCK21 | 5260341 | 297 | 191561 | 76 | ecoli[ST-131] | ecoli[ADK-53,FUMC-40,GYRB-47,ICD-13,MDH-36,PURA-28,RECA-29] | blaCTX-M-15 | 41 |
| urine | NCK22 | 5316624 | 404 | 162977 | 67 | ecoli[ST-131] | ecoli[ADK-53,FUMC-40,GYRB-47,ICD-13,MDH-36,PURA-28,RECA-29] | blaCTX-M-27 | 41 |
| urine | NCK23 | 5355317 | 234 | 191567 | 70 | ecoli[ST-131] | ecoli[ADK-53,FUMC-40,GYRB-47,ICD-13,MDH-36,PURA-28,RECA-29] | blaCTX-M-15 | 30 |
| urine | NCK24 | 5010163 | 159 | 222098 | 115 | ecoli[ST-1193] | ecoli[ADK-14,FUMC-14,GYRB-10,ICD-200,MDH-17,PURA-7,RECA-10] | blaCTX-M-27 | 64 |
| urine | NCK25 | 5353691 | 265 | 184915 | 110 | ecoli[ST-38] | ecoli[ADK-4,FUMC-26,GYRB-2,ICD-25,MDH-5,PURA-5,RECA-19] | blaCTX-M-15 | ND |
| urine | NCK26 | 5488156 | 336 | 191413 | 114 | ecoli[ST-131] | ecoli[ADK-53,FUMC-40,GYRB-47,ICD-13,MDH-36,PURA-28,RECA-29] | blaCTX-M-15 | 30 |
| urine | NCK27 | 4946177 | 190 | 193118 | 107 | ecoli[ST-131] | ecoli[ADK-53,FUMC-40,GYRB-47,ICD-13,MDH-36,PURA-28,RECA-29] | blaCTX-M-14 | 41 |
| urine | NCK3 | 5070020 | 242 | 147550 | 97 | ecoli[ST-38] | ecoli[ADK-4,FUMC-26,GYRB-2,ICD-25,MDH-5,PURA-5,RECA-19] | blaCTX-M-14 | 5 |
| urine | NCK30 | 5375218 | 300 | 159079 | 86 | ecoli[ST-6309] | ecoli[ADK-53,FUMC-830,GYRB-47,ICD-13,MDH-36,PURA-28,RECA-29] | blaCTX-M-15 | 30 |
| urine | NCK32 | 5207721 | 254 | 224128 | 78 | ecoli[ST-372] | ecoli[ADK-88,FUMC-103,GYRB-19,ICD-36,MDH-23,PURA-44,RECA-26] | blaCTX-M-1 | 102 |
| urine | NCK33 | 5370489 | 490 | 122886 | 74 | ecoli[ST-38] | ecoli[ADK-4,FUMC-26,GYRB-2,ICD-25,MDH-5,PURA-5,RECA-19] | blaCTX-M-15 | ND |
| urine | NCK36 | 5406101 | 502 | 161613 | 68 | ecoli[ST-404] | ecoli[ADK-14,FUMC-14,GYRB-10,ICD-14,MDH-17,PURA-7,RECA-74] | blaCTX-M-14 | 27 |
| urine | NCK38 | 5166475 | 284 | 260763 | 136 | ecoli[ST-73] | ecoli[ADK-36,FUMC-24,GYRB-9,ICD-13,MDH-17,PURA-11,RECA-25] | blaCTX-M-14 | 30 |
| urine | NCK39 | 5539535 | 688 | 108378 | 41 | ecoli[ST-38] | ecoli[ADK-4,FUMC-26,GYRB-2,ICD-25,MDH-5,PURA-5,RECA-19] | blaCTX-M-27 | ND |
| urine | NCK4 | 5371274 | 384 | 114517 | 99 | ecoli[ST-405] | ecoli[ADK-35,FUMC-37,GYRB-29,ICD-25,MDH-4,PURA-5,RECA-73] | blaCTX-M-1 | 27 |
| urine | NCK41 | 5158616 | 165 | 225817 | 136 | ecoli[ST-1193] | ecoli[ADK-14,FUMC-14,GYRB-10,ICD-200,MDH-17,PURA-7,RECA-10] | blaCTX-M-15 | 64 |
| urine | NCK42 | 5236685 | 455 | 198638 | 93 | ecoli[ST-95] | ecoli[ADK-37,FUMC-38,GYRB-19,ICD-37,MDH-17,PURA-11,RECA-26] | blaCTX-M-14 | 27 |
| urine | NCK43 | 5385661 | 618 | 321772 | 96 | ecoli[ST-394] | ecoli[ADK-21,FUMC-35,GYRB-61,ICD-52,MDH-5,PURA-5,RECA-4] | blaCTX-M-15 | 30 |
| urine | NCK5 | 5178244 | 269 | 158059 | 72 | ecoli[ST-131] | ecoli[ADK-53,FUMC-40,GYRB-47,ICD-13,MDH-36,PURA-28,RECA-29] | blaCTX-M-15 | 41 |
| urine | NCK8 | 5454497 | 339 | 162738 | 87 | ecoli[ST-117] | ecoli[ADK-20,FUMC-45,GYRB-41,ICD-43,MDH-5,PURA-32,RECA-2] | blaCTX-M-1 | 97 |
| urine | NCK9 | 5452372 | 529 | 137702 | 95 | ecoli[ST-69] | ecoli[ADK-21,FUMC-35,GYRB-27,ICD-6,MDH-5,PURA-5,RECA-4] | blaCTX-M-15 | 27 |
| urine | NIC10 | 5265187 | 423 | 103151 | 60 | ecoli[ST-38] | ecoli[ADK-4,FUMC-26,GYRB-2,ICD-25,MDH-5,PURA-5,RECA-19] | blaCTX-M-14 | 65 |
| urine | NIC3 | 5295939 | 592 | 163985 | 88 | ecoli[ST-69] | ecoli[ADK-21,FUMC-35,GYRB-27,ICD-6,MDH-5,PURA-5,RECA-4] | blaCTX-M-15 | 27 |
| urine | NIC4 | 4901081 | 132 | 200675 | 261 | ecoli[ST-1147] | ecoli[ADK-6,FUMC-4,GYRB-4,ICD-18,MDH-135,PURA-7,RECA-6] | blaCTX-M-15 | 25 |
| urine | NIC5 | 5412391 | 308 | 148698 | 57 | ecoli[ST-131] | ecoli[ADK-53,FUMC-40,GYRB-47,ICD-13,MDH-36,PURA-28,RECA-29] | blaCTX-M-15 | 30 |
| urine | RDE10 | 5276733 | 264 | 156225 | 109 | ecoli[ST-131] | ecoli[ADK-53,FUMC-40,GYRB-47,ICD-13,MDH-36,PURA-28,RECA-29] | blaCTX-M-15 | 27 |
| urine | RDE14 | 5402214 | 334 | 135592 | 127 | ecoli[ST-38] | ecoli[ADK-4,FUMC-26,GYRB-2,ICD-25,MDH-5,PURA-5,RECA-19] | blaCTX-M-15 | 5 |
| urine | RDE15 | 5030551 | 249 | 106422 | 83 | ecoli[ST-58] | ecoli[ADK-6,FUMC-4,GYRB-4,ICD-16,MDH-24,PURA-8,RECA-14] | blaCTX-M-1 | 34 |
| urine | RDE16 | 5111572 | 242 | 163301 | 81 | ecoli[ST-131] | ecoli[ADK-53,FUMC-40,GYRB-47,ICD-13,MDH-36,PURA-28,RECA-29] | blaCTX-M-27 | 30 |
| urine | RDE2 | 5302351 | 498 | 146863 | 103 | ecoli[ST-69] | ecoli[ADK-21,FUMC-35,GYRB-27,ICD-6,MDH-5,PURA-5,RECA-4] | blaCTX-M-14 | 27 |
| urine | RDE3 | 5336895 | 332 | 123408 | 86 | ecoli[ST-38] | ecoli[ADK-4,FUMC-26,GYRB-2,ICD-25,MDH-5,PURA-5,RECA-19] | blaCTX-M-14 | 5 |
| urine | RDE4 | 5132418 | 378 | 163094 | 115 | ecoli[ST-1982] | ecoli[ADK-53,FUMC-40,GYRB-254,ICD-13,MDH-36,PURA-28,RECA-29] | blaCTX-M-3 | 41 |
| urine | RDE5 | 5157590 | 249 | 191446 | 51 | ecoli[ST-131] | ecoli[ADK-53,FUMC-40,GYRB-47,ICD-13,MDH-36,PURA-28,RECA-29] | blaCTX-M-27 | 41 |
| urine | RDE6 | 5306552 | 301 | 162973 | 136 | ecoli[ST-131] | ecoli[ADK-53,FUMC-40,GYRB-47,ICD-13,MDH-36,PURA-28,RECA-29] | blaCTX-M-3 | 27 |
| urine | RDE7 | 5201207 | 235 | 203165 | 185 | ecoli[ST-131] | ecoli[ADK-53,FUMC-40,GYRB-47,ICD-13,MDH-36,PURA-28,RECA-29] | blaCTX-M-15 | 30 |
| urine | RDE8 | 5245659 | 217 | 192393 | 90 | ecoli[ST-131] | ecoli[ADK-53,FUMC-40,GYRB-47,ICD-13,MDH-36,PURA-28,RECA-29] | blaCTX-M-15 | 30 |
| urine | RDE9 | 5129383 | 159 | 335378 | 66 | ecoli[ST-88] | ecoli[ADK-6,FUMC-4,GYRB-12,ICD-1,MDH-20,PURA-12,RECA-7] | blaCTX-M-1 | 39-like |
| urine | RDP10 | 5386541 | 406 | 124368 | 85 | ecoli[ST-2020] | ecoli[ADK-21,FUMC-35,GYRB-27,ICD-6,MDH-220,PURA-5,RECA-4] | blaCTX-M-15 | 27 |
| urine | RDP13 | 5209673 | 203 | 203949 | 264 | ecoli[ST-131] | ecoli[ADK-53,FUMC-40,GYRB-47,ICD-13,MDH-36,PURA-28,RECA-29] | blaCTX-M-15 | 30 |
| urine | RDP15 | 5125792 | 223 | 234970 | 112 | ecoli[ST-95] | ecoli[ADK-37,FUMC-38,GYRB-19,ICD-37,MDH-17,PURA-11,RECA-26] | blaCTX-M-1 | 30 |
| urine | RDP17 | 5255893 | 212 | 213293 | 164 | ecoli[ST-131] | ecoli[ADK-53,FUMC-40,GYRB-47,ICD-13,MDH-36,PURA-28,RECA-29] | blaCTX-M-27 | 41 |
| urine | RDP19 | 5579372 | 392 | 145254 | 166 | ecoli[ST-219] | ecoli[ADK-58,FUMC-53,GYRB-53,ICD-58,MDH-24,PURA-1,RECA-42] | blaCTX-M-55 | 370 |
| urine | RDP2 | 5241846 | 415 | 104232 | 151 | ecoli[ST-12] | ecoli[ADK-13,FUMC-13,GYRB-9,ICD-13,MDH-16,PURA-10,RECA-9] | blaSHV-12 | 5 |
| urine | RDP20 | 5286316 | 263 | 174694 | 102 | ecoli[ST-131] | ecoli[ADK-53,FUMC-40,GYRB-47,ICD-13,MDH-36,PURA-28,RECA-29] | blaCTX-M-15 | 30 |
| urine | RDP23 | 5285390 | 367 | 123200 | 93 | ecoli[ST-38] | ecoli[ADK-4,FUMC-26,GYRB-2,ICD-25,MDH-5,PURA-5,RECA-19] | blaCTX-M-15 | 5 |
| urine | RDP26 | 5342571 | 357 | 218599 | 69 | ecoli[ST-73] | ecoli[ADK-36,FUMC-24,GYRB-9,ICD-13,MDH-17,PURA-11,RECA-25] | blaCTX-M-15 | 10 |
| urine | RDP28 | 5343607 | 312 | 136184 | 90 | ecoli[ST-131] | ecoli[ADK-53,FUMC-40,GYRB-47,ICD-13,MDH-36,PURA-28,RECA-29] | blaCTX-M-14 | 30 |
| urine | RDP30 | 5060143 | 211 | 154939 | 85 | ecoli[ST-131] | ecoli[ADK-53,FUMC-40,GYRB-47,ICD-13,MDH-36,PURA-28,RECA-29] | blaCTX-M-27 | 30 |
| urine | RDP32 | 5293461 | 390 | 209436 | 322 | ecoli[ST-69] | ecoli[ADK-21,FUMC-35,GYRB-27,ICD-6,MDH-5,PURA-5,RECA-4] | blaCTX-M-1 | 27 |
| urine | RDP33 | 5179892 | 212 | 191752 | 86 | ecoli[ST-95] | ecoli[ADK-37,FUMC-38,GYRB-19,ICD-37,MDH-17,PURA-11,RECA-26] | blaCTX-M-15 | 27 |
| urine | RDP34 | 5340774 | 300 | 156287 | 106 | ecoli[ST-131] | ecoli[ADK-53,FUMC-40,GYRB-47,ICD-13,MDH-36,PURA-28,RECA-29] | blaCTX-M-15 | 30 |
| urine | RDP4 | 5224916 | 256 | 193338 | 116 | ecoli[ST-131] | ecoli[ADK-53,FUMC-40,GYRB-47,ICD-13,MDH-36,PURA-28,RECA-29] | blaCTX-M-15 | 30 |
| urine | ROU11 | 5309849 | 261 | 232247 | 98 | ecoli[ST-12] | ecoli[ADK-13,FUMC-13,GYRB-9,ICD-13,MDH-16,PURA-10,RECA-9] | blaCTX-M-15 | 27 |
| urine | ROU12 | 5218731 | 223 | 191421 | 90 | ecoli[ST-131] | ecoli[ADK-53,FUMC-40,GYRB-47,ICD-13,MDH-36,PURA-28,RECA-29] | blaCTX-M-15 | 30 |
| urine | ROU13 | 5334892 | 236 | 173829 | 141 | ecoli[ST-131] | ecoli[ADK-53,FUMC-40,GYRB-47,ICD-13,MDH-36,PURA-28,RECA-29] | blaCTX-M-15 | 30 |
| urine | ROU15 | 5441558 | 526 | 111499 | 182 | ecoli[ST-38] | ecoli[ADK-4,FUMC-26,GYRB-2,ICD-25,MDH-5,PURA-5,RECA-19] | blaCTX-M-27 | ND |
| urine | ROU16 | 5202011 | 233 | 206719 | 93 | ecoli[ST-131] | ecoli[ADK-53,FUMC-40,GYRB-47,ICD-13,MDH-36,PURA-28,RECA-29] | blaCTX-M-27 | 41 |
| urine | ROU17 | 5039159 | 150 | 319339 | 118 | ecoli[ST-127] | ecoli[ADK-13,FUMC-14,GYRB-19,ICD-36,MDH-23,PURA-11,RECA-10] | blaCTX-M-15 | 180 |
| urine | ROU20 | 5295636 | 246 | 191405 | 202 | ecoli[ST-131] | ecoli[ADK-53,FUMC-40,GYRB-47,ICD-13,MDH-36,PURA-28,RECA-29] | blaCTX-M-15 | 30 |
| urine | ROU3 | 5286745 | 288 | 135703 | 79 | ecoli[ST-131] | ecoli[ADK-53,FUMC-40,GYRB-47,ICD-13,MDH-36,PURA-28,RECA-29] | blaCTX-M-15 | 30 |
| urine | ROU4 | 5121299 | 345 | 140173 | 86 | ecoli[ST-73] | ecoli[ADK-36,FUMC-24,GYRB-9,ICD-13,MDH-17,PURA-11,RECA-25] | blaCTX-M-14 | 30 |
| urine | ROU6 | 5000117 | 153 | 229723 | 101 | ecoli[ST-362] | ecoli[ADK-62,FUMC-100,GYRB-17,ICD-31,MDH-5,PURA-5,RECA-4] | blaCTX-M-15 | 96 |
| urine | ROU9 | 5127002 | 230 | 145583 | 115 | ecoli[ST-75] | ecoli[ADK-6,FUMC-6,GYRB-5,ICD-10,MDH-20,PURA-23,RECA-6] | blaCTX-M-1 | 35 |
| urine | RSA1 | 5035860 | 183 | 149948 | 102 | ecoli[ST-131] | ecoli[ADK-53,FUMC-40,GYRB-47,ICD-13,MDH-36,PURA-28,RECA-29] | blaCTX-M-27 | 30 |
| urine | RSA10 | 5216390 | 258 | 171088 | 80 | ecoli[ST-131] | ecoli[ADK-53,FUMC-40,GYRB-47,ICD-13,MDH-36,PURA-28,RECA-29] | blaCTX-M-15 | 30 |
| urine | RSA11 | 5053899 | 310 | 186303 | 83 | ecoli[ST-127] | ecoli[ADK-13,FUMC-14,GYRB-19,ICD-36,MDH-23,PURA-11,RECA-10] | blaCTX-M-3 | 2 |
| urine | RSA13 | 5487921 | 540 | 171768 | 89 | ecoli[ST-38] | ecoli[ADK-4,FUMC-26,GYRB-2,ICD-25,MDH-5,PURA-5,RECA-19] | blaCTX-M-27 | ND |
| urine | RSA16 | 5172224 | 256 | 156071 | 97 | ecoli[ST-131] | ecoli[ADK-53,FUMC-40,GYRB-47,ICD-13,MDH-36,PURA-28,RECA-29] | blaCTX-M-15 | 27 |
| urine | RSA17 | 5129825 | 339 | 121281 | 99 | ecoli[ST-167] | ecoli[ADK-10,FUMC-11,GYRB-4,ICD-8,MDH-8,PURA-13,RECA-2] | blaCTX-M-1 | 215 |
| urine | RSA18 | 4940942 | 161 | 167726 | 94 | ecoli[ST-101] | ecoli[ADK-43,FUMC-41,GYRB-15,ICD-18,MDH-11,PURA-7,RECA-6] | blaSHV-12 | 86 |
| urine | RSA20 | 4957531 | 213 | 128707 | 110 | ecoli[Unknown ST] | ecoli[ADK-6,FUMC-4,GYRB-102,ICD-16,MDH-24,PURA-8,RECA-14] | blaCTX-M-1 | 32 |
| urine | RSA4 | 4883860 | 284 | 110972 | 89 | ecoli[ST-761] | ecoli[ADK-10,FUMC-11,GYRB-5,ICD-8,MDH-8,PURA-8,RECA-2] | blaCTX-M-1 | 27 |
| urine | RSA6 | 5348399 | 333 | 161803 | 91 | ecoli[ST-141] | ecoli[ADK-13,FUMC-52,GYRB-10,ICD-14,MDH-17,PURA-25,RECA-17] | blaCTX-M-14 | 5 |
| urine | RSA8 | 5073324 | 288 | 131447 | 71 | ecoli[ST-616] | ecoli[ADK-6,FUMC-4,GYRB-12,ICD-16,MDH-24,PURA-8,RECA-14] | blaCTX-M-1 | 121 |
| urine | SCA1 | 5259714 | 192 | 191458 | 114 | ecoli[ST-131] | ecoli[ADK-53,FUMC-40,GYRB-47,ICD-13,MDH-36,PURA-28,RECA-29] | blaCTX-M-15 | 30 |
| urine | SCA4 | 5030200 | 272 | 191438 | 74 | ecoli[ST-131] | ecoli[ADK-53,FUMC-40,GYRB-47,ICD-13,MDH-36,PURA-28,RECA-29] | blaCTX-M-14 | 41 |
| urine | SCA5 | 4997480 | 208 | 159284 | 48 | ecoli[ST-1982] | ecoli[ADK-53,FUMC-40,GYRB-254,ICD-13,MDH-36,PURA-28,RECA-29] | blaCTX-M-27 | 30 |
| urine | SCA6 | 5188452 | 215 | 162933 | 93 | ecoli[ST-131] | ecoli[ADK-53,FUMC-40,GYRB-47,ICD-13,MDH-36,PURA-28,RECA-29] | blaCTX-M-15 | 30 |
| urine | SCA8 | 5126721 | 183 | 268413 | 103 | ecoli[ST-95] | ecoli[ADK-37,FUMC-38,GYRB-19,ICD-37,MDH-17,PURA-11,RECA-26] | blaCTX-M-14 | 30 |
| urine | SCA9 | 5222605 | 360 | 191564 | 81 | ecoli[ST-95] | ecoli[ADK-37,FUMC-38,GYRB-19,ICD-37,MDH-17,PURA-11,RECA-26] | blaCTX-M-15 | 27 |
| faeces | 162005CD | 5544717 | 572 | 88963 | 53 | ecoli[ST-62] | ecoli[ADK-28,FUMC-33,GYRB-25,ICD-29,MDH-7,PURA-11,RECA-24] | blaCTX-M-14 | 44 |
| faeces | 162010CD | 5224838 | 364 | 157379 | 95 | ecoli[ST-131] | ecoli[ADK-53,FUMC-40,GYRB-47,ICD-13,MDH-36,PURA-28,RECA-29] | blaCTX-M-27 | 30 |
| faeces | 162025CD | 5152303 | 304 | 213081 | 128 | ecoli[ST-131] | ecoli[ADK-53,FUMC-40,GYRB-47,ICD-13,MDH-36,PURA-28,RECA-29] | blaCTX-M-27 | 41 |
| faeces | 162048CD | 4943461 | 250 | 163362 | 98 | ecoli[ST-602] | ecoli[ADK-6,FUMC-19,GYRB-33,ICD-26,MDH-11,PURA-8,RECA-6] | blaCTX-M-15 | 86 |
| faeces | 162081CD | 5325066 | 673 | 121573 | 73 | ecoli[ST-295] | ecoli[ADK-6,FUMC-4,GYRB-12,ICD-1,MDH-9,PURA-2,RECA-7] | blaCTX-M-14 | 54 |
| faeces | 162082CD1 | 5259209 | 528 | 58186 | 35 | ecoli[ST-59] | ecoli[ADK-27,FUMC-32,GYRB-24,ICD-29,MDH-26,PURA-19,RECA-22] | blaCTX-M-1 | 41 |
| faeces | 162082CD3 | 5270322 | 486 | 246315 | 148 | ecoli[ST-404] | ecoli[ADK-14,FUMC-14,GYRB-10,ICD-14,MDH-17,PURA-7,RECA-74] | blaCTX-M-1 | 27 |
| faeces | 162091CD | 5362438 | 413 | 147020 | 88 | ecoli[ST-5150] | ecoli[ADK-4,FUMC-26,GYRB-2,ICD-501,MDH-5,PURA-5,RECA-19] | blaCTX-M-55 | 65 |
| faeces | 162096CD | 5423527 | 453 | 146758 | 88 | ecoli[ST-394] | ecoli[ADK-21,FUMC-35,GYRB-61,ICD-52,MDH-5,PURA-5,RECA-4] | blaCTX-M-14 | 47 |
| faeces | 162121CD | 5080928 | 281 | 109026 | 66 | ecoli[ST-6338] | ecoli[ADK-290,FUMC-54,GYRB-55,ICD-352,MDH-514,PURA-323,RECA-38] | blaCTX-M-14 | 453-like |
| faeces | 162199CD | 5042799 | 187 | 192788 | 116 | ecoli[ST-131] | ecoli[ADK-53,FUMC-40,GYRB-47,ICD-13,MDH-36,PURA-28,RECA-29] | blaCTX-M-27 | 30 |
| faeces | 162206CD | 5383693 | 296 | 181591 | 109 | ecoli[ST-131] | ecoli[ADK-53,FUMC-40,GYRB-47,ICD-13,MDH-36,PURA-28,RECA-29] | blaCTX-M-15 | 30 |
| faeces | 162231CD | 5051014 | 185 | 215060 | 129 | ecoli[ST-69] | ecoli[ADK-21,FUMC-35,GYRB-27,ICD-6,MDH-5,PURA-5,RECA-4] | blaCTX-M-15 | 27 |
| faeces | 162271CD | 5225825 | 243 | 139430 | 84 | ecoli[ST-131] | ecoli[ADK-53,FUMC-40,GYRB-47,ICD-13,MDH-36,PURA-28,RECA-29] | blaCTX-M-15 | 30 |
| faeces | 162311CD | 5164790 | 224 | 201181 | 121 | ecoli[ST-131] | ecoli[ADK-53,FUMC-40,GYRB-47,ICD-13,MDH-36,PURA-28,RECA-29] | blaCTX-M-27 | 41 |
| faeces | 162332CD1 | 4925551 | 110 | 290509 | 175 | ecoli[ST-224] | ecoli[ADK-6,FUMC-4,GYRB-33,ICD-16,MDH-11,PURA-8,RECA-6] | blaCTX-M-1 | 61 |
| faeces | 162332CD2 | 4635218 | 210 | 105015 | 63 | ecoli[ST-746] | ecoli[ADK-10,FUMC-7,GYRB-4,ICD-8,MDH-12,PURA-8,RECA-2] | blaCTX-M-1 | 54 |
| faeces | 162372CD | 5044282 | 194 | 173822 | 104 | ecoli[ST-131] | ecoli[ADK-53,FUMC-40,GYRB-47,ICD-13,MDH-36,PURA-28,RECA-29] | blaCTX-M-27 | 30 |
| faeces | 162386CD | 5383489 | 559 | 170365 | 102 | ecoli[ST-131] | ecoli[ADK-53,FUMC-40,GYRB-47,ICD-13,MDH-36,PURA-28,RECA-29] | blaCTX-M-15 | 30 |
| faeces | 162407CD | 5096037 | 240 | 173690 | 104 | ecoli[ST-131] | ecoli[ADK-53,FUMC-40,GYRB-47,ICD-13,MDH-36,PURA-28,RECA-29] | blaCTX-M-27 | 30 |
| faeces | 162411CD | 5035474 | 202 | 235431 | 141 | ecoli[ST-131] | ecoli[ADK-53,FUMC-40,GYRB-47,ICD-13,MDH-36,PURA-28,RECA-29] | blaCTX-M-15 | 41 |
| faeces | 162444CD | 4999176 | 222 | 207753 | 125 | ecoli[ST-69] | ecoli[ADK-21,FUMC-35,GYRB-27,ICD-6,MDH-5,PURA-5,RECA-4] | blaCTX-M-15 | 27 |
| faeces | 162461CD | 5466842 | 517 | 191578 | 115 | ecoli[ST-131] | ecoli[ADK-53,FUMC-40,GYRB-47,ICD-13,MDH-36,PURA-28,RECA-29] | blaCTX-M-15 | 30 |
| faeces | 162464CD | 5446299 | 500 | 182503 | 110 | ecoli[ST-38] | ecoli[ADK-4,FUMC-26,GYRB-2,ICD-25,MDH-5,PURA-5,RECA-19] | blaCTX-M-27 | ND |
| faeces | 162472CD | 5263616 | 332 | 145258 | 87 | ecoli[ST-38] | ecoli[ADK-4,FUMC-26,GYRB-2,ICD-25,MDH-5,PURA-5,RECA-19] | blaCTX-M-27 | 65 |
| faeces | 162502CD | 5166853 | 351 | 246733 | 148 | ecoli[ST-131] | ecoli[ADK-53,FUMC-40,GYRB-47,ICD-13,MDH-36,PURA-28,RECA-29] | blaCTX-M-27 | 41 |
| faeces | 162505CD | 5395174 | 348 | 228227 | 137 | ecoli[ST-141] | ecoli[ADK-13,FUMC-52,GYRB-10,ICD-14,MDH-17,PURA-25,RECA-17] | blaCTX-M-14 | 5 |
| faeces | 162520CD | 5345623 | 481 | 225849 | 136 | ecoli[ST-131] | ecoli[ADK-53,FUMC-40,GYRB-47,ICD-13,MDH-36,PURA-28,RECA-29] | blaCTX-M-15 | 30 |
| faeces | 162526CD | 5530657 | 627 | 175639 | 106 | ecoli[ST-131] | ecoli[ADK-53,FUMC-40,GYRB-47,ICD-13,MDH-36,PURA-28,RECA-29] | blaCTX-M-15 | 30 |
| faeces | 162581CD | 4996353 | 199 | 186035 | 112 | ecoli[ST-349] | ecoli[ADK-34,FUMC-36,GYRB-39,ICD-87,MDH-67,PURA-16,RECA-4] | blaCTX-M-15 | 27 |
| faeces | 162603CD | 4665088 | 238 | 144261 | 87 | ecoli[ST-10] | ecoli[ADK-10,FUMC-11,GYRB-4,ICD-8,MDH-8,PURA-8,RECA-2] | blaCTX-M-15 | 54 |
| faeces | 162604CD | 5169509 | 220 | 174886 | 105 | ecoli[ST-131] | ecoli[ADK-53,FUMC-40,GYRB-47,ICD-13,MDH-36,PURA-28,RECA-29] | blaCTX-M-27 | 30 |
| faeces | 162645CD | 5433978 | 372 | 152175 | 91 | ecoli[ST-57] | ecoli[ADK-6,FUMC-31,GYRB-5,ICD-28,MDH-1,PURA-1,RECA-2] | blaSHV-12 | 27 |
| faeces | 162654CD | 5140912 | 257 | 345641 | 208 | ecoli[ST-73] | ecoli[ADK-36,FUMC-24,GYRB-9,ICD-13,MDH-17,PURA-11,RECA-25] | blaCTX-M-14 | 103-like |
| faeces | 162727CD | 5233106 | 548 | 175740 | 106 | ecoli[ST-95] | ecoli[ADK-37,FUMC-38,GYRB-19,ICD-37,MDH-17,PURA-11,RECA-26] | blaCTX-M-14 | 27 |
| faeces | L1 | 4981259 | 188 | 189056 | 105 | ecoli[ST-23] | ecoli[ADK-6,FUMC-4,GYRB-12,ICD-1,MDH-20,PURA-13,RECA-7] | blaCTX-M-1 | 35 |
| faeces | L102 | 5444162 | 149 | 200915 | 270 | ecoli[ST-131] | ecoli[ADK-53,FUMC-40,GYRB-47,ICD-13,MDH-36,PURA-28,RECA-29] | blaCTX-M-15 | 30 |
| faeces | L102b | 5282044 | 340 | 175911 | 151 | ecoli[ST-131] | ecoli[ADK-53,FUMC-40,GYRB-47,ICD-13,MDH-36,PURA-28,RECA-29] | blaCTX-M-15 | 30 |
| faeces | L103 | 5059877 | 761 | 9478 | 30 | ecoli[ST-131] | ecoli[ADK-53,FUMC-40,GYRB-47,ICD-13,MDH-36,PURA-28,RECA-29] | blaCTX-M-15 | 30 |
| faeces | L106 | 4998206 | 177 | 55273 | 34 | ecoli[ST-131] | ecoli[ADK-53,FUMC-40,GYRB-47,ICD-13,MDH-36,PURA-28,RECA-29] | blaCTX-M-27 | 30 |
| faeces | L110 | 4899611 | 648 | 11248 | 36 | ecoli[Unknown ST] | ecoli[ADK-53,FUMC-40,GYRB-47,ICD-13,MDH-36,PURA-28,RECA-109] | blaCTX-M-14 | 41 |
| faeces | L111 | 5309315 | 113 | 131328 | 50 | ecoli[ST-131] | ecoli[ADK-53,FUMC-40,GYRB-47,ICD-13,MDH-36,PURA-28,RECA-29] | blaCTX-M-15 | 30 |
| faeces | L113 | 5046227 | 152 | 279155 | 111 | ecoli[ST-1268] | ecoli[ADK-35,FUMC-22,GYRB-16,ICD-6,MDH-168,PURA-5,RECA-95] | blaCTX-M-1 | 27 |
| faeces | L114 | 5201866 | 199 | 58914 | 75 | ecoli[ST-131] | ecoli[ADK-53,FUMC-40,GYRB-47,ICD-13,MDH-36,PURA-28,RECA-29] | blaCTX-M-15 | 30 |
| faeces | L116 | 5041843 | 190 | 184447 | 87 | ecoli[ST-131] | ecoli[ADK-53,FUMC-40,GYRB-47,ICD-13,MDH-36,PURA-28,RECA-29] | blaCTX-M-27 | 30 |
| faeces | L117 | 5024286 | 254 | 181622 | 75 | ecoli[ST-23] | ecoli[ADK-6,FUMC-4,GYRB-12,ICD-1,MDH-20,PURA-13,RECA-7] | blaCTX-M-1 | 35 |
| faeces | L120 | 5359245 | 522 | 179477 | 123 | ecoli[ST-117] | ecoli[ADK-20,FUMC-45,GYRB-41,ICD-43,MDH-5,PURA-32,RECA-2] | blaCTX-M-14 | 97 |
| faeces | L121 | 5170171 | 208 | 261480 | 81 | ecoli[ST-69] | ecoli[ADK-21,FUMC-35,GYRB-27,ICD-6,MDH-5,PURA-5,RECA-4] | blaCTX-M-1 | 483 |
| faeces | L122 | 5346431 | 353 | 173587 | 70 | ecoli[ST-131] | ecoli[ADK-53,FUMC-40,GYRB-47,ICD-13,MDH-36,PURA-28,RECA-29] | blaCTX-M-15 | 30 |
| faeces | L123 | 5340478 | 571 | 130699 | 81 | ecoli[ST-117] | ecoli[ADK-20,FUMC-45,GYRB-41,ICD-43,MDH-5,PURA-32,RECA-2] | blaCTX-M-14 | 97 |
| faeces | L124 | 5327273 | 319 | 159680 | 69 | ecoli[ST-131] | ecoli[ADK-53,FUMC-40,GYRB-47,ICD-13,MDH-36,PURA-28,RECA-29] | blaCTX-M-1 | 41 |
| faeces | L126 | 5023051 | 166 | 191564 | 99 | ecoli[ST-131] | ecoli[ADK-53,FUMC-40,GYRB-47,ICD-13,MDH-36,PURA-28,RECA-29] | blaCTX-M-27 | 30 |
| faeces | L127 | 5530752 | 849 | 79600 | 75 | ecoli[ST-301] | ecoli[ADK-78,FUMC-27,GYRB-5,ICD-10,MDH-12,PURA-8,RECA-2] | blaCTX-M-1 | 54 |
| faeces | L134 | 5025562 | 82 | 167155 | 62 | ecoli[ST-131] | ecoli[ADK-53,FUMC-40,GYRB-47,ICD-13,MDH-36,PURA-28,RECA-29] | blaCTX-M-14 | 30 |
| faeces | L135 | 5274928 | 378 | 162643 | 93 | ecoli[ST-131] | ecoli[ADK-53,FUMC-40,GYRB-47,ICD-13,MDH-36,PURA-28,RECA-29] | blaCTX-M-15 | 30 |
| faeces | L136 | 5033930 | 86 | 181166 | 64 | ecoli[ST-131] | ecoli[ADK-53,FUMC-40,GYRB-47,ICD-13,MDH-36,PURA-28,RECA-29] | blaCTX-M-27 | 30 |
| faeces | L14 | 5374577 | 106 | 176250 | 77 | ecoli[ST-131] | ecoli[ADK-53,FUMC-40,GYRB-47,ICD-13,MDH-36,PURA-28,RECA-29] | blaCTX-M-15 | 30 |
| faeces | L140 | 5062160 | 87 | 149540 | 44 | ecoli[ST-131] | ecoli[ADK-53,FUMC-40,GYRB-47,ICD-13,MDH-36,PURA-28,RECA-29] | blaCTX-M-15 | 30 |
| faeces | L146 | 5015861 | 83 | 191577 | 77 | ecoli[ST-131] | ecoli[ADK-53,FUMC-40,GYRB-47,ICD-13,MDH-36,PURA-28,RECA-29] | blaCTX-M-27 | 30 |
| faeces | L148 | 5118496 | 114 | 158975 | 72 | ecoli[ST-131] | ecoli[ADK-53,FUMC-40,GYRB-47,ICD-13,MDH-36,PURA-28,RECA-29] | blaCTX-M-27 | 30 |
| faeces | L149 | 5123620 | 103 | 198762 | 80 | ecoli[ST-131] | ecoli[ADK-53,FUMC-40,GYRB-47,ICD-13,MDH-36,PURA-28,RECA-29] | blaCTX-M-27 | 30 |
| faeces | L150 | 5101321 | 99 | 156353 | 40 | ecoli[ST-131] | ecoli[ADK-53,FUMC-40,GYRB-47,ICD-13,MDH-36,PURA-28,RECA-29] | blaCTX-M-27 | 41 |
| faeces | L155 | 5043610 | 90 | 192018 | 71 | ecoli[ST-131] | ecoli[ADK-53,FUMC-40,GYRB-47,ICD-13,MDH-36,PURA-28,RECA-29] | blaCTX-M-27 | 30 |
| faeces | L162 | 5058115 | 76 | 233366 | 63 | ecoli[ST-131] | ecoli[ADK-53,FUMC-40,GYRB-47,ICD-13,MDH-36,PURA-28,RECA-29] | blaCTX-M-27 | 41 |
| faeces | L166 | 5012481 | 86 | 184243 | 62 | ecoli[ST-131] | ecoli[ADK-53,FUMC-40,GYRB-47,ICD-13,MDH-36,PURA-28,RECA-29] | blaCTX-M-27 | 30 |
| faeces | L168 | 5032624 | 97 | 199399 | 65 | ecoli[ST-131] | ecoli[ADK-53,FUMC-40,GYRB-47,ICD-13,MDH-36,PURA-28,RECA-29] | blaCTX-M-15 | 41 |
| faeces | L169 | 5115065 | 100 | 158984 | 70 | ecoli[ST-131] | ecoli[ADK-53,FUMC-40,GYRB-47,ICD-13,MDH-36,PURA-28,RECA-29] | blaCTX-M-27 | 99 |
| faeces | L172 | 4997822 | 83 | 221127 | 52 | ecoli[ST-131] | ecoli[ADK-53,FUMC-40,GYRB-47,ICD-13,MDH-36,PURA-28,RECA-29] | blaCTX-M-27 | 42 |
| faeces | L173 | 5044339 | 101 | 99587 | 40 | ecoli[ST-131] | ecoli[ADK-53,FUMC-40,GYRB-47,ICD-13,MDH-36,PURA-28,RECA-29] | blaCTX-M-15 | 30 |
| faeces | L175 | 5174720 | 94 | 191011 | 56 | ecoli[ST-131] | ecoli[ADK-53,FUMC-40,GYRB-47,ICD-13,MDH-36,PURA-28,RECA-29] | blaCTX-M-27 | 30 |
| faeces | L176 | 5266660 | 83 | 218314 | 84 | ecoli[ST-131] | ecoli[ADK-53,FUMC-40,GYRB-47,ICD-13,MDH-36,PURA-28,RECA-29] | blaCTX-M-15 | 30 |
| faeces | L177 | 4928532 | 155 | 154465 | 99 | ecoli[ST-131] | ecoli[ADK-53,FUMC-40,GYRB-47,ICD-13,MDH-36,PURA-28,RECA-29] | blaCTX-M-14 | 30 |
| faeces | L178 | 5402350 | 709 | 127450 | 87 | ecoli[ST-38] | ecoli[ADK-4,FUMC-26,GYRB-2,ICD-25,MDH-5,PURA-5,RECA-19] | blaCTX-M-15 | 5 |
| faeces | L179 | 5118280 | 377 | 118899 | 87 | ecoli[ST-10] | ecoli[ADK-10,FUMC-11,GYRB-4,ICD-8,MDH-8,PURA-8,RECA-2] | blaCTX-M-14 | 27 |
| faeces | L180 | 6306917 | 314 | 235445 | 117 | ecoli[Unknown ST] | ecoli[ADK-212,FUMC-677,GYRB-311,ICD-507,MDH-636,PURA-15,RECA-32] | blaCTX-M-14 | ND |
| faeces | L181 | 5449245 | 474 | 143115 | 81 | ecoli[ST-38] | ecoli[ADK-4,FUMC-26,GYRB-2,ICD-25,MDH-5,PURA-5,RECA-19] | blaCTX-M-15 | 5 |
| faeces | L182 | 4865014 | 139 | 219881 | 153 | ecoli[ST-349] | ecoli[ADK-34,FUMC-36,GYRB-39,ICD-87,MDH-67,PURA-16,RECA-4] | blaCTX-M-65 | 93 |
| faeces | L183 | 5247858 | 315 | 224267 | 105 | ecoli[ST-1623] | ecoli[ADK-214,FUMC-19,GYRB-12,ICD-16,MDH-9,PURA-8,RECA-7] | blaCTX-M-3 | ND |
| faeces | L184 | 4979935 | 170 | 255068 | 153 | ecoli[ST-457] | ecoli[ADK-101,FUMC-88,GYRB-97,ICD-108,MDH-26,PURA-79,RECA-2] | blaCTX-M-14 | 145 |
| faeces | L185 | 5382248 | 303 | 207197 | 120 | ecoli[ST-414] | ecoli[ADK-18,FUMC-22,GYRB-20,ICD-23,MDH-5,PURA-15,RECA-4] | blaCTX-M-14 | ND |
| faeces | L186 | 4711528 | 116 | 197391 | 117 | ecoli[ST-348] | ecoli[ADK-6,FUMC-29,GYRB-32,ICD-16,MDH-11,PURA-8,RECA-14] | blaCTX-M-1 | 32 |
| faeces | L187 | 5301075 | 245 | 191819 | 99 | ecoli[ST-38] | ecoli[ADK-4,FUMC-26,GYRB-2,ICD-25,MDH-5,PURA-5,RECA-19] | blaCTX-M-15 | ND |
| faeces | L188 | 4923816 | 189 | 199689 | 102 | ecoli[ST-23] | ecoli[ADK-6,FUMC-4,GYRB-12,ICD-1,MDH-20,PURA-13,RECA-7] | blaCTX-M-1 | 35 |
| faeces | L189 | 5158388 | 326 | 90149 | 99 | ecoli[ST-93] | ecoli[ADK-6,FUMC-11,GYRB-4,ICD-10,MDH-7,PURA-8,RECA-6] | blaCTX-M-1 | ND |
| faeces | L190 | 5257765 | 376 | 167298 | 84 | ecoli[ST-3172] | ecoli[ADK-14,FUMC-14,GYRB-10,ICD-14,MDH-272,PURA-92,RECA-10] | blaCTX-M-15 | 30 |
| faeces | L191 | 5196412 | 198 | 191998 | 96 | ecoli[ST-131] | ecoli[ADK-53,FUMC-40,GYRB-47,ICD-13,MDH-36,PURA-28,RECA-29] | blaCTX-M-15 | 30 |
| faeces | L192 | 5376428 | 318 | 170357 | 114 | ecoli[ST-131] | ecoli[ADK-53,FUMC-40,GYRB-47,ICD-13,MDH-36,PURA-28,RECA-29] | blaCTX-M-15 | 30 |
| faeces | L193 | 5350086 | 271 | 153987 | 186 | ecoli[ST-117] | ecoli[ADK-20,FUMC-45,GYRB-41,ICD-43,MDH-5,PURA-32,RECA-2] | blaSHV-12 | 97 |
| faeces | L194 | 5125071 | 641 | 105101 | 91 | ecoli[ST-93] | ecoli[ADK-6,FUMC-11,GYRB-4,ICD-10,MDH-7,PURA-8,RECA-6] | blaCTX-M-14 | 32 |
| faeces | L195 | 4783902 | 170 | 131263 | 256 | ecoli[ST-10] | ecoli[ADK-10,FUMC-11,GYRB-4,ICD-8,MDH-8,PURA-8,RECA-2] | blaCTX-M-1 | 54 |
| faeces | L197 | 5223171 | 180 | 159165 | 139 | ecoli[ST-131] | ecoli[ADK-53,FUMC-40,GYRB-47,ICD-13,MDH-36,PURA-28,RECA-29] | blaCTX-M-27 | 30 |
| faeces | L198 | 5331357 | 302 | 173037 | 71 | ecoli[ST-131] | ecoli[ADK-53,FUMC-40,GYRB-47,ICD-13,MDH-36,PURA-28,RECA-29] | blaCTX-M-15 | 30 |
| faeces | L199 | 5486781 | 528 | 150234 | 109 | ecoli[ST-38] | ecoli[ADK-4,FUMC-26,GYRB-2,ICD-25,MDH-5,PURA-5,RECA-19] | blaCTX-M-27 | ND |
| faeces | L201 | 5137043 | 241 | 201978 | 120 | ecoli[ST-69] | ecoli[ADK-21,FUMC-35,GYRB-27,ICD-6,MDH-5,PURA-5,RECA-4] | blaCTX-M-14 | 27 |
| faeces | L202 | 4652461 | 260 | 86987 | 123 | ecoli[ST-6856] | ecoli[ADK-6,FUMC-11,GYRB-4,ICD-732,MDH-8,PURA-8,RECA-2] | blaCTX-M-14 | ND |
| faeces | L203 | 5044481 | 178 | 173819 | 107 | ecoli[ST-131] | ecoli[ADK-53,FUMC-40,GYRB-47,ICD-13,MDH-36,PURA-28,RECA-29] | blaCTX-M-27 | 30 |
| faeces | L204 | 5366364 | 272 | 162884 | 118 | ecoli[ST-131] | ecoli[ADK-53,FUMC-40,GYRB-47,ICD-13,MDH-36,PURA-28,RECA-29] | blaCTX-M-14 | 30 |
| faeces | L205 | 5186454 | 440 | 141561 | 100 | ecoli[ST-130] | ecoli[ADK-18,FUMC-22,GYRB-20,ICD-6,MDH-5,PURA-5,RECA-4] | blaCTX-M-15 | 54 |
| faeces | L206 | 5371935 | 213 | 173449 | 150 | ecoli[ST-131] | ecoli[ADK-53,FUMC-40,GYRB-47,ICD-13,MDH-36,PURA-28,RECA-29] | blaCTX-M-14 | 30 |
| faeces | L207 | 4993308 | 269 | 135422 | 43 | ecoli[ST-23] | ecoli[ADK-6,FUMC-4,GYRB-12,ICD-1,MDH-20,PURA-13,RECA-7] | blaCTX-M-14 | 35 |
| faeces | L208 | 5288353 | 208 | 191567 | 121 | ecoli[ST-131] | ecoli[ADK-53,FUMC-40,GYRB-47,ICD-13,MDH-36,PURA-28,RECA-29] | blaCTX-M-15 | 30 |
| faeces | L209 | 5157969 | 229 | 287749 | 102 | ecoli[ST-127] | ecoli[ADK-13,FUMC-14,GYRB-19,ICD-36,MDH-23,PURA-11,RECA-10] | blaCTX-M-1 | 310 |
| faeces | L211 | 5075356 | 160 | 204684 | 109 | ecoli[ST-108] | ecoli[ADK-46,FUMC-36,GYRB-37,ICD-25,MDH-5,PURA-16,RECA-33] | blaCTX-M-14 | 48 |
| faeces | L212 | 5163299 | 201 | 191437 | 184 | ecoli[ST-131] | ecoli[ADK-53,FUMC-40,GYRB-47,ICD-13,MDH-36,PURA-28,RECA-29] | blaCTX-M-27 | 30 |
| faeces | L213 | 5061716 | 298 | 364309 | 161 | ecoli[ST-95] | ecoli[ADK-37,FUMC-38,GYRB-19,ICD-37,MDH-17,PURA-11,RECA-26] | blaCTX-M-14 | 41 |
| faeces | L214 | 5319359 | 361 | 274013 | 134 | ecoli[ST-69] | ecoli[ADK-21,FUMC-35,GYRB-27,ICD-6,MDH-5,PURA-5,RECA-4] | blaCTX-M-1 | 27 |
| faeces | L215 | 5197075 | 222 | 345761 | 103 | ecoli[ST-73] | ecoli[ADK-36,FUMC-24,GYRB-9,ICD-13,MDH-17,PURA-11,RECA-25] | blaCTX-M-15 | 9 |
| faeces | L216 | 5231308 | 263 | 180492 | 155 | ecoli[ST-95] | ecoli[ADK-37,FUMC-38,GYRB-19,ICD-37,MDH-17,PURA-11,RECA-26] | blaCTX-M-15 | 27 |
| faeces | L217 | 5062202 | 153 | 261044 | 143 | ecoli[ST-69] | ecoli[ADK-21,FUMC-35,GYRB-27,ICD-6,MDH-5,PURA-5,RECA-4] | blaSHV-12 | 483 |
| faeces | L218 | 5044353 | 150 | 191457 | 121 | ecoli[ST-131] | ecoli[ADK-53,FUMC-40,GYRB-47,ICD-13,MDH-36,PURA-28,RECA-29] | blaCTX-M-27 | 30 |
| faeces | L219 | 4902259 | 216 | 115148 | 124 | ecoli[ST-940] | ecoli[ADK-6,FUMC-6,GYRB-22,ICD-16,MDH-11,PURA-1,RECA-7] | blaCTX-M-15 | ND |
| faeces | L221 | 5039280 | 169 | 177197 | 136 | ecoli[ST-2197] | ecoli[ADK-10,FUMC-11,GYRB-4,ICD-8,MDH-8,PURA-219,RECA-174] | blaSHV-12 | 23 |
| faeces | L222 | 5120343 | 240 | 223397 | 48 | ecoli[ST-12] | ecoli[ADK-13,FUMC-13,GYRB-9,ICD-13,MDH-16,PURA-10,RECA-9] | blaCTX-M-15 | 5 |
| faeces | L224 | 5222323 | 173 | 173785 | 184 | ecoli[ST-131] | ecoli[ADK-53,FUMC-40,GYRB-47,ICD-13,MDH-36,PURA-28,RECA-29] | blaCTX-M-27 | 30 |
| faeces | L225 | 5021549 | 177 | 255132 | 190 | ecoli[ST-457] | ecoli[ADK-101,FUMC-88,GYRB-97,ICD-108,MDH-26,PURA-79,RECA-2] | blaCTX-M-14 | 145 |
| faeces | L227 | 5147251 | 188 | 191433 | 107 | ecoli[ST-131] | ecoli[ADK-53,FUMC-40,GYRB-47,ICD-13,MDH-36,PURA-28,RECA-29] | blaCTX-M-27 | 30 |
| faeces | L229 | 5478868 | 397 | 143534 | 160 | ecoli[ST-38] | ecoli[ADK-4,FUMC-26,GYRB-2,ICD-25,MDH-5,PURA-5,RECA-19] | blaCTX-M-15 | 5 |
| faeces | L230 | 5151002 | 228 | 278537 | 162 | ecoli[ST-127] | ecoli[ADK-13,FUMC-14,GYRB-19,ICD-36,MDH-23,PURA-11,RECA-10] | blaCTX-M-1 | 310 |
| faeces | L232 | 5294549 | 274 | 154499 | 81 | ecoli[ST-131] | ecoli[ADK-53,FUMC-40,GYRB-47,ICD-13,MDH-36,PURA-28,RECA-29] | blaCTX-M-14 | 30 |
| faeces | L24 | 5152602 | 143 | 110194 | 30 | ecoli[ST-131] | ecoli[ADK-53,FUMC-40,GYRB-47,ICD-13,MDH-36,PURA-28,RECA-29] | blaCTX-M-1 | 41 |
| faeces | L25 | 5038744 | 726 | 10189 | 75 | ecoli[ST-131] | ecoli[ADK-53,FUMC-40,GYRB-47,ICD-13,MDH-36,PURA-28,RECA-29] | blaCTX-M-15 | 30 |
| faeces | L26 | 4868711 | 137 | 137788 | 114 | ecoli[ST-58] | ecoli[ADK-6,FUMC-4,GYRB-4,ICD-16,MDH-24,PURA-8,RECA-14] | blaCTX-M-1 | 32 |
| faeces | L271 | 5436218 | 423 | 134960 | 119 | ecoli[ST-394] | ecoli[ADK-21,FUMC-35,GYRB-61,ICD-52,MDH-5,PURA-5,RECA-4] | blaCTX-M-14 | ND |
| faeces | L273 | 5230447 | 631 | 36458 | 61 | ecoli[Unknown ST] | ecoli[ADK-27,FUMC-32,GYRB-24,ICD-29,MDH-127,PURA-19,RECA-22] | blaCTX-M-1 | 41 |
| faeces | L274 | 5224204 | 273 | 117822 | 129 | ecoli[ST-131] | ecoli[ADK-53,FUMC-40,GYRB-47,ICD-13,MDH-36,PURA-28,RECA-29] | blaCTX-M-15 | 30 |
| faeces | L275 | 5168305 | 224 | 159770 | 91 | ecoli[ST-131] | ecoli[ADK-53,FUMC-40,GYRB-47,ICD-13,MDH-36,PURA-28,RECA-29] | blaCTX-M-27 | 30 |
| faeces | L276 | 4876555 | 263 | 119823 | 96 | ecoli[ST-6361] | ecoli[ADK-577,FUMC-297,GYRB-260,ICD-254,MDH-1,PURA-2,RECA-2] | blaCTX-M-27 | 31 |
| faeces | L277 | 5228229 | 329 | 135169 | 98 | ecoli[ST-38] | ecoli[ADK-4,FUMC-26,GYRB-2,ICD-25,MDH-5,PURA-5,RECA-19] | blaCTX-M-27 | 65 |
| faeces | L278 | 5395627 | 369 | 221980 | 96 | ecoli[ST-141] | ecoli[ADK-13,FUMC-52,GYRB-10,ICD-14,MDH-17,PURA-25,RECA-17] | blaCTX-M-14 | 5 |
| faeces | L29 | 5293556 | 325 | 204694 | 90 | ecoli[ST-1640] | ecoli[ADK-6,FUMC-31,GYRB-4,ICD-28,MDH-1,PURA-1,RECA-2] | blaCTX-M-1 | 31 |
| faeces | L31 | 5021424 | 105 | 148680 | 60 | ecoli[ST-131] | ecoli[ADK-53,FUMC-40,GYRB-47,ICD-13,MDH-36,PURA-28,RECA-29] | blaCTX-M-14 | 30 |
| faeces | L35 | 5070017 | 88 | 137798 | 35 | ecoli[ST-131] | ecoli[ADK-53,FUMC-40,GYRB-47,ICD-13,MDH-36,PURA-28,RECA-29] | blaCTX-M-15 | 30 |
| faeces | L36 | 5057383 | 149 | 93200 | 33 | ecoli[ST-131] | ecoli[ADK-53,FUMC-40,GYRB-47,ICD-13,MDH-36,PURA-28,RECA-29] | blaCTX-M-15 | 30 |
| faeces | L39 | 4999483 | 139 | 106221 | 30 | ecoli[ST-131] | ecoli[ADK-53,FUMC-40,GYRB-47,ICD-13,MDH-36,PURA-28,RECA-29] | blaCTX-M-14 | 41 |
| faeces | L41 | 5037047 | 598 | 13023 | 82 | ecoli[Unknown ST] | ecoli[ADK-1,FUMC-40,GYRB-47,ICD-13,MDH-36,PURA-28,RECA-29] | blaCTX-M-1 | 41 |
| faeces | L49 | 5029768 | 374 | 24353 | 87 | ecoli[Unknown ST] | ecoli[ADK-53,FUMC-40,GYRB-47,ICD-13,MDH-36,PURA-28,RECA-109] | blaCTX-M-1 | 41 |
| faeces | L50 | 5371911 | 488 | 171313 | 122 | ecoli[ST-117] | ecoli[ADK-20,FUMC-45,GYRB-41,ICD-43,MDH-5,PURA-32,RECA-2] | blaCTX-M-1 | 97 |
| faeces | L51 | 4998524 | 162 | 248320 | 96 | ecoli[ST-131] | ecoli[ADK-53,FUMC-40,GYRB-47,ICD-13,MDH-36,PURA-28,RECA-29] | blaCTX-M-27 | 41 |
| faeces | L52 | 5128356 | 184 | 183547 | 117 | ecoli[ST-1193] | ecoli[ADK-14,FUMC-14,GYRB-10,ICD-200,MDH-17,PURA-7,RECA-10] | blaCTX-M-15 | 64 |
| faeces | L58 | 5068658 | 90 | 164180 | 70 | ecoli[ST-131] | ecoli[ADK-53,FUMC-40,GYRB-47,ICD-13,MDH-36,PURA-28,RECA-29] | blaCTX-M-27 | 30 |
| faeces | L6 | 5203267 | 357 | 160168 | 89 | ecoli[ST-38] | ecoli[ADK-4,FUMC-26,GYRB-2,ICD-25,MDH-5,PURA-5,RECA-19] | blaCTX-M-14 | 5 |
| faeces | L60 | 5071231 | 260 | 37077 | 27 | ecoli[ST-131] | ecoli[ADK-53,FUMC-40,GYRB-47,ICD-13,MDH-36,PURA-28,RECA-29] | blaCTX-M-27 | 30 |
| faeces | L62 | 4614480 | 1097 | 5689 | 112 | ecoli[ST-131] | ecoli[ADK-53,FUMC-40,GYRB-47,ICD-13,MDH-36,PURA-28,RECA-29] | blaCTX-M-27 | 30 |
| faeces | L64 | 5078938 | 483 | 18851 | 68 | ecoli[ST-131] | ecoli[ADK-53,FUMC-40,GYRB-47,ICD-13,MDH-36,PURA-28,RECA-29] | blaCTX-M-27 | 30 |
| faeces | L70 | 5029354 | 355 | 24081 | 104 | ecoli[ST-131] | ecoli[ADK-53,FUMC-40,GYRB-47,ICD-13,MDH-36,PURA-28,RECA-29] | blaCTX-M-27 | 30 |
| faeces | L72 | 5613335 | 1177 | 35469 | 122 | ecoli[ST-349] | ecoli[ADK-34,FUMC-36,GYRB-39,ICD-87,MDH-67,PURA-16,RECA-4] | blaCTX-M-15 | 54 |
| faeces | L78 | 5052298 | 346 | 207990 | 99 | ecoli[ST-367] | ecoli[ADK-6,FUMC-4,GYRB-12,ICD-1,MDH-20,PURA-65,RECA-7] | blaCTX-M-1 | 27 |
| faeces | L79 | 4991802 | 165 | 208074 | 100 | ecoli[ST-367] | ecoli[ADK-6,FUMC-4,GYRB-12,ICD-1,MDH-20,PURA-65,RECA-7] | blaCTX-M-1 | 27 |
| faeces | L82 | 5189089 | 364 | 228821 | 121 | ecoli[ST-73] | ecoli[ADK-36,FUMC-24,GYRB-9,ICD-13,MDH-17,PURA-11,RECA-25] | blaCTX-M-14 | 30 |
| faeces | L88 | 5096216 | 342 | 30292 | 32 | ecoli[ST-131] | ecoli[ADK-53,FUMC-40,GYRB-47,ICD-13,MDH-36,PURA-28,RECA-29] | blaCTX-M-15 | 30 |
| faeces | L89 | 5129653 | 164 | 112161 | 55 | ecoli[Unknown ST] | ecoli[ADK-53,FUMC-798,GYRB-47,ICD-13,MDH-36,PURA-28,RECA-29] | blaCTX-M-15 | 30 |
| faeces | L90 | 5185987 | 402 | 287697 | 156 | ecoli[ST-73] | ecoli[ADK-36,FUMC-24,GYRB-9,ICD-13,MDH-17,PURA-11,RECA-25] | blaCTX-M-14 | 30 |
| faeces | L92 | 5210276 | 340 | 162781 | 92 | ecoli[ST-349] | ecoli[ADK-34,FUMC-36,GYRB-39,ICD-87,MDH-67,PURA-16,RECA-4] | blaCTX-M-15 | 54 |
| faeces | L93 | 5320630 | 378 | 204835 | 117 | ecoli[ST-1640] | ecoli[ADK-6,FUMC-31,GYRB-4,ICD-28,MDH-1,PURA-1,RECA-2] | blaCTX-M-1 | 31 |

**Figure S1:** ROC curve of *papGII* and/or *traJ* combination used as a test for “high risk of FUTI” ESBL-Ec
